# Phenotypic plasticity of polyploid plant species promotes transgressive behaviour in their hybrids

**DOI:** 10.1093/aobpla/ply055

**Published:** 2018-09-23

**Authors:** Blanca Gallego-Tévar, Alfredo E Rubio-Casal, Alfonso de Cires, Enrique Figueroa, Brenda J Grewell, Jesús M Castillo

**Affiliations:** 1 Departamento de Biología Vegetal y Ecología, Universidad de Sevilla, Ap, Sevilla, Spain; 2 USDA-ARS Invasive Species and Pollinator Health Unit, University of California, Davis, CA, USA

**Keywords:** Abiotic stress, cordgrass, halophyte, heterosis, hybridization, hybrid vigour, invasive plant, *Spartina*

## Abstract

Hybridization is a frequent process that leads to relevant evolutionary consequences, but there is a lack of studies regarding the relationships of the variability of the response of parental plant species to environmental gradients and the responses of their hybrids at a phenotypic level. We designed an experiment in which we exposed two reciprocal cordgrass hybrids, *Spartina maritima* × *densiflora* and *S. densiflora* × *maritima*, and their parental species to four salinity concentrations for 30 days. The main objectives were to compare the performance of the hybrids with that of their parents, to distinguish the phenotypic inheritance operating in the hybrids and to analyse the relationships between the variability in the responses of the parents and the responses of their hybrids to salinity. We characterized the responses and the degree of variability for 37 foliar traits. Both hybrids presented greater salinity tolerance than their parents, showing their highest percentage of transgressive traits at both extremes of the salinity gradient. When the parental plants themselves showed a more plastic response for a given trait, there was a greater chance that their hybrid developed a transgressive behaviour for this trait. This finding supports a new focus to be applied for the artificial development of vigorous hybrid crops.

## Teaser

This study reports the fitness and the phenotypic inheritance of two wild invasive *Spartina* (cordgrass) hybrids in relation to their parental species along a wide estuarine salinity gradient. The hybrids outperformed their parental species showing greater fitness and some transgressive traits. Plant traits displaying greater variability in the polyploid parents led to a higher number of transgressive responses in the hybrids. This work is the continuation of our research on the tolerance of halophytes to abiotic stresses and their phenotypic plasticity, now adding an evolutionary perspective to the process of hybridization.

## Introduction

Hybridization is a frequent process in both plants and animals that leads to relevant evolutionary and ecological consequences (Arnold 1992). The ecological performance of hybrids depends on the expression of genes that control traits related to their stress tolerance and fitness (Chen 2013). Thus, the novel genotypes obtained by hybridization commonly exhibit phenotypic traits with intermediate values between both parents due to an additive genetic control when the traits are controlled by a large number of genes that act independently, as well as similar to parental species as a product of a dominant inheritance (Favre and Karrenberg 2011). But hybrids can also display another phenotypic inheritance that produces phenotypic traits outside the ranges of variability of both parental species, showing transgressive phenotypes as a product of heterosis or hybrid vigour (Rieseberg *et al.* 1999). In wild invasive hybrids, fixed heterosis leads to an increase of invasiveness (Ellstrand and Schierenbeck 2000) as they may be fitter than the parental species and able to colonize, establish and tolerate more extreme environments (Vilà *et al.* 2000; Castillo *et al.* 2010; Hovick and Whitney 2014; Parepa *et al.* 2014). In cultivated hybrids, heterosis is being applied in crop production to develop more vigorous and better performing cultivars (Fu *et al.* 2014). The molecular mechanisms underlying heterosis have been subject of long deliberations (Chen 2010, 2013; Baranwal *et al.* 2012). In addition to the genomic mechanisms, recent studies have revealed the importance of epigenetic changes in key genes regulating fitness-related traits in hybrids (Salmon *et al.* 2005; Ni *et al.* 2009). Additionally, there is evidence that a greater genetic differentiation between parents results in greater heterosis in their hybrids (East 1936; Chen 2010).

On the other hand, hybridization has been associated with high phenotypic plasticity (Ainouche and Jenczewski 2010; Te Beest *et al.* 2012; Cara *et al.* 2013) that also contributes to the enhanced ability of hybrids to occupy wide ecological ranges. Phenotypic plasticity can be adaptive when it is regulated by heritable mechanisms (Matesanz *et al.* 2010) and, therefore, evolve itself as an independent functional trait. In hybrids and allopolyploids, variations in the expected additive or parental-like phenotypic plasticity are controlled by changes in genes expression both at transcriptional and post-transcriptional levels (Jackson and Chen 2011). Furthermore, phenotypic plasticity can be also regulated at an epigenetic level (Parepa *et al.* 2014). However, phenotypic inheritance and phenotypic plasticity are both regulated at genetic and epigenetic levels, but there is a lack of studies regarding the relationships of the variability on the response of parental species to environmental gradients and the inheritance at work in their hybrids.

Cordgrasses (former genus *Spartina*) are halophytes with a wide distribution all around the world (Strong and Ayres 2013). All *Spartina* species are polyploids (Ainouche *et al.* 2012) and hybridization has repeatedly occurred between them, playing an important role in their evolution (Ainouche *et al.* 2009). Recently, two first-generation (F_1_) reciprocal hybrids between the native European *Spartina maritima* (2n = 6x = 60) from low salt marshes and the invasive South American *Spartina densiflora* (2n = 7x = 70) from middle marshes have been described on the Gulf of Cadiz (Southwest Iberian Peninsula). *Spartina maritima* × *densiflora* has ca. 65 chromosomes, with *S. maritima* as the maternal species, and this hybrid occurs in low marshes. In contrast, *S. densiflora* × *maritima* has ca. 95 chromosomes, with *S. densiflora* as the maternal species, and it colonizes middle marshes (Castillo *et al.* 2010). Both F_1_ hybrids are sterile and show some transgressive behaviours such as higher growth rates and taller tillers than their parental species (Castillo *et al.* 2010). Given the differences in performance of *S. maritima*, *S. densiflora* and their hybrids, these taxa constitute a good model for studying how parental responses along an environmental gradient determine the phenotypic inheritance operating in their hybrids. With this aim, we designed a glasshouse experiment in which *S. maritima* × *densiflora*, *S. densiflora* × *maritima* and both parental species were exposed to four salinity concentrations (from freshwater to hypersalinity). To evaluate their responses and degree of response variability, we measured 37 distinct plant traits. Our main objectives were to: (i) compare the performance of the hybrids with that of their parents by evaluating the magnitude and variability of plant trait responses to salinity; (ii) distinguish the phenotypic inheritance operating in the hybrids; and (iii) analyse the relationships between the variability in the plant trait responses of the parents and the responses of their hybrids. Our hypothesis was that the *Spartina* hybrids would outperform their parental species showing greater fitness, especially at the extreme salinities, due to heterosis. Additionally, we postulated that traits showing greater variability in the parents would lead to a higher number of transgressive responses in the hybrids given the greater possibilities of advantageous combinations.

## Methods

### Plant collection and experimental design

The *S. densiflora* × *maritima* hybrid (2n = ca. 65) is the result of the fecundation of the reduced ovule of *S. densiflora* (2n = 7x = 70) by a reduced gamete of *S. maritima* (2n = 6x = 60). The hybrid *S. maritima* × *densiflora* (2n = ca. 95) has *S. maritima* as a maternal species contributing with its total genome (unreduced gamete) and a reduced gamete of *S. densiflora* (Castillo *et al.* 2010). Below ground biomass (BGB) of five different individuals of *S. maritima* (*Sm*), *S. densiflora* (*Sd*) and both of their hybrids (*Sm*×*d* and *Sd*×*m*) were collected from natural populations in the Special Area of Conservation San Bruno Marsh (Guadiana River Estuary, Huelva, Spain; 37°10ʹ37°16ʹN, 7°28ʹ–7°16ʹW) in November 2015. Parental species were differentiated in the field by morphology following Mobberley (1953). Hybrids were also initially distinguished based on morphological expression, and we have confirmed identification using chloroplast and nuclear DNA, and ploidy assessments (per Castillo *et al.* 2010). Collections of individual plants were separated by a minimum of 2 m to ensure sampling of discrete individuals. *Spartina maritima* and the hybrid *S. maritima* × *densiflora* were collected from the low marsh and *S. densiflora* and *S. densiflora* × *maritima* from the middle marsh, where they were more abundant. Collected plant material was transported to the greenhouse facility of the University of Seville where it was cleaned and trimmed, with roots removed. Rhizomes were then weighed and potted in 16 cm diameter × 15 cm high pots using expanded perlite (Comercial Projar S.A., Valencia, Spain) as a substrate. Rhizomes weights were as similar as possible between taxa, being 4 ± 1 g for *S. maritima* due to its long and sparse rhizomes, 9 ± 1 g for *S. densiflora* due to their short and dense rhizomes and intermediate (7 ± 1 g) for their hybrids.

The pots were arranged in groups of six in 38.5 cm wide × 53.5 cm long × 5.0 cm deep black plastic trays, and then submerged in water with liquid fertiliser (Naturplant, Fertilizantes Orgánicos Melguizo, S.L., Seville, Spain) to a depth of 4 cm for acclimatization and growth for 4 months. Subsequently, the same five genotypes (individuals) of each taxon collected in natural populations were divided into four pieces of rhizomes from which experimental plants were obtained and were randomly placed in four salinity treatments ranging from freshwater to hypersalinity (0.5, 10, 20 and 40 ppt salinity) using sea salt Instant Ocean® (Aquarium Systems Inc., Mentor, OH, USA) plus 20 % Hoagland’s nutrient solution which was changed weekly. Opaque plastic black bags were used to cover those areas of the trays not occupied by pots in order to prevent algae proliferation in the solution. The highest salinity treatments were established by increasing salinity by 10 ppt each week until reaching the final concentration to avoid osmotic shock. The experiment lasted 30 days in April–May 2016 in the glasshouse with a controlled temperature of 21–25 °C and natural sunlight.

### Data collection

Mechanistic and functional plant traits were measured after 30 days of salinity treatments to assess responses of the *Spartina* hybrids and their parental species to different salinity levels. Foliar measurements standardized by always using the youngest, completely unfolded adult leaf to avoid differences due to the ontogeny of leaves. Measured plant traits were all foliar traits of the studied *Spartina* taxa since we expected to see significant changes across the experimental salinity gradient because the leaf (the organ of photosynthesis and transpiration) is highly sensitive to salt stress (Suárez 2011).

#### Leaf morphology

We quantified leaf area and specific leaf area (SLA), because these traits may change with salinity as morphological acclimations to salt stress (Castillo *et al.* 2014). Leaf area was calculated as the triangle area obtained with the leaf base width and its length, both measured using a ruler. Specific leaf area (m^−2^ g^−1^) was calculated as the ratio between the leaf area and its dry weight (Garnier *et al*. 2001). Sub-replicates of three leaves per plant were conducted for these measurements.

#### Leaf biochemistry

Leaf samples for biochemical analyses were always collected within 2 h of solar noon. Leaf water content (LWC) was determined for one leaf per plant as LWC (%) = (FW − DW) × 100/FW, where FW was the fresh weight and DW was the dry weight after oven-drying samples at 80 °C for 48 h (Castillo *et al.* 2007).

Free proline content in leaves was recorded as an indicator of salt stress (Grewell *et al.* 2016). It was determined for one leaf per plant following the procedure presented in Bates *et al.* (1973)**[see****Supporting Information—Methods S1****]**.

Malondialdehyde (MDA) is a product of lipid peroxidation, so its leaf content was recorded as an indicator of oxidative stress (Meloni *et al.* 2003). Leaf MDA content was measured for one leaf per plant according to the method described in Dhindsa *et al.* (1981)**[see****Supporting Information—Methods S2****]**.

Photosynthetic pigments were measured from one leaf per plant according to the method described in Arnon (1949) and Lichtenthaler (1987)**[see****Supporting Information—Methods S3****]**. Photosynthetic pigments have been used to indicate the effects of salt stress in the photosynthetic apparatus of *Spartina* species (Castillo *et al.* 2014; Grewell *et al.* 2016). The ratios Chl (*a* + *b*):Car and Chl *a*:Chl *b* were calculated. Additionally, the relative content (%) of the non-photosynthetic anthocyanin pigments was also recorded measuring the absorbance at 530 nm using the same spectrophotometer (Mancinelli *et al.* 1975; Khlestkina *et al.* 2014). Anthocyanin accumulation may provide antioxidant and photoprotection functions, and act as a dehydration-tolerance mechanism under salt stress (Chalker-Scott 1999; Lee and Collins 2001; Gould *et al.* 2002).

Dry leaf tissue from one leaf per plant was ground to pass through a No. 4 mesh sieve prior to measurement of total carbon (C) and nitrogen (N) content using a Perkin Elmer 2400 CHNS/O analyzer (Perkin Elmer, Waltham, MA, USA). C:N ratio was calculated as an indicator of salt stress (Yousef and Sprent 1983).

#### Salt excretion

In order to measure the salt exudation rate from leaves, two flag leaves per plant were marked and rinsed with deionized water to remove salt exuded previously. After 48 h, leaf tissue of known area from the marked leaves was placed into a vial with 3 mL of deionized water, and the vial was shaken to dissolve all salt that had accumulated on the leaf surface. Dissolved salts were then measured with a portable conductivity meter (Crison-522, Hach Lange Spain S.L.U., Barcelona, Spain) and excretion rate was calculated.

#### Chlorophyll fluorescence

Chl fluorescence is a useful tool to assess the effects of salt stress on the photosynthetic apparatus (Castillo *et al.* 2005).

Therefore, light- and dark-adapted Chl fluorescence were measured for five leaves per plant at sunrise (at 12 °C, 60 % air relative humidity and a photosynthetic photon flux density (PPFD) of 20 µmol photon m^–2^ s^–1^) and at noon (at 26 °C, 45 % air relative humidity and PPFD of 1450 µmol photon m^–2^ s^–1^) with a portable modulated fluorimeter (FMS-2, Hansatech Instruments Ltd, Norfolk, UK) using leaf clips for dark adaptation for 30 min. Chl fluorescence parameters were measured according to Maxwell and Johnson (2000) and Schreiber *et al.* (1986)**[see****Supporting Information—Methods S4****]**. In addition, delayed Chl fluorescence was measured using a modular optical imaging system (NightSHADE LB985, Berthold Technologies GmbH & Co., Baden-Württemberg, Germany) to quantify the post-illumination luminescence emitted by Chl *a*, mainly by PSII that is an indicator of its photochemistry (reviewed in Jursinic 1986).

#### Gas exchange

Gas exchange measurements were obtained by using an infrared gas analyzer in an open system (LI-6400, Li-COR Inc., Lincoln, NE, USA) and using a Clark type oxygen electrode (Leaflab 2 System, Hansatech Instruments Ltd, Norfolk, UK). The first was used to determine net photosynthesis rate (*A*), stomatal conductance to CO_2_ (*G*_s_) and intracellular CO_2_ concentration (*C*_i_) at fixed 400 ppt CO_2_ concentration, 15–20 °C, 36.5 ± 1.3 % relative humidity, a PPFD of 1000 μmol m^−2^ s^−1^ and a flow rate set to 350 μmol s^−1^ within 2 h of solar noon. Water use efficiency (WUE; mmol CO_2_ per mol H_2_O) was calculated from simultaneous measures of photosynthesis rate and stomatal conductance. The oxygen electrode was employed to measure maximum photosynthesis rate (*A*_max_) by providing a saturated atmosphere of CO_2_ with a 1 M carbonate/bicarbonate buffer (pH 9.0) at PPFD of 1200–1400 µmol photon m^–2^ s^–1^ and 25 °C. Net photosynthesis rate and *G*_s_ are optimal indicators of the effects of salt stress on CO_2_ fixation and the water use, respectively (Chaves *et al.* 2009).

#### Leaf growth

Apical leaf growth (mm day^−1^) is a measure of plant fitness. It was quantified by applying red permanent sealer to base of the youngest leaf and the top of three tillers per plant to measure the separation between the two plants parts 48 h later (Castillo *et al.* 2014).

### Data analyses

#### Phenotypic inheritance

Inheritance operating in the hybrids *S. maritima* × *densiflora* and *S. densiflora* × *maritima* was analysed for the above-mentioned 37 plant traits related to their response to salt stress. Three types of phenotypic inheritance were distinguished (Favre and Karrenberg 2011). First, dominant inheritance (D) was considered when a hybrid showed a trait similar to one of its parents (D-*Sm* for *S. maritima*; D-*Sd* for *S. densiflora*); parental codominance (D-*Sm*,*Sd*) was considered when a trait of a hybrid was similar to both parents. Second, parental additivity (I) was recorded when a trait for a hybrid was intermediate between significantly different values of its parents. The third type of phenotypic inheritance, transgressive phenotype (T), corresponded with a trait of a hybrid being different from both parental species (outperforming both parental species at least in ±5 %). The three inheritance types were quantified for each variable and at each salinity level for both hybrids at the population level (*S. maritima* × *densiflora* at low marshes and *S. densiflora* × *maritima* at middle marshes), and for each population at the individual level.

#### Trait variability and fitness

Inter-treatment trait variability index was used as a general indicator of trait variability among salinity levels and individuals for a given taxon. It was calculated for each taxon including all salinity treatments as the ratio (in percentage) of the difference between the maximum (*X*_max_) and the minimum (*X*_min_) values of a given trait divided by the maximum (Valladares *et al.* 2006).

$$\text{Inter}.\text{ Trait Var}.=[(X_{max}-X_{min})/X_{max}]*100$$

Mean intra-population trait variability index indicated the intrinsic variability between individuals of the same population, since the same genotypes (individuals) of each taxon were used at each salinity treatment. It was calculated as the arithmetic mean (*n* = 4 salinity treatments) of the ratios (in percentage) of the differences between the maximum (*x*_max_) and the minimum (*x*_min_) values divided by the maximum of a given trait for a taxon in a certain salinity (Castillo *et al.* 2018).

$$\text{Intra}.\text{ Trait Var}.\text{ =}\frac{\sum_{i=1}^{4}[(x_{max}-x_{min})/x_{max}]*100}{4}$$

Phenotypic plasticity index (PPI) for a given trait and taxon was obtained after subtracting the mean intra-population trait variability from the inter-population trait variability. In this way, just the variability associated with trait difference related to salt stress was obtained, removing the variation due to intrinsic individual differences that seems to be especially relevant for hybrid and polyploid taxa (Te Beest *et al.* 2012) such as the four studied *Spartina* (Ainouche *et al.* 2012).

Fitness (%) was calculated for each taxon at each salinity as the population average of the percentages (*x*_*i*_) of six key physiological functional traits and growth (*F*_v_/*F*_m_, Φ_PSII_ at both sunrise and noon, *A*, and leaf growth) in relation to the maximum population value (*x*_max_) at any salinity (Dodd 2005).

$$Fitness=\overset{5}{\underset{i=1}{\sum }}\frac{x_{i}*100}{x_{max}}$$

#### Statistics

Deviations of all data were calculated as the standard error of the mean (SE). All statistical analyses were carried out using Sigma-Plot for Windows version 14.0 applying a significance level (α) of 0.05 for every analysis. Two-way analysis of variance (ANOVA) with taxon and salinity as grouping factors was conducted to compare mean values for each plant trait and the fitness, and one-way ANOVA with taxon as a grouping factor to compare the three trait variability indexes. Prior to the use of the parametric models, data series were tested for normality with the Kolmogorov–Smirnov’s test and for homogeneity of variance with the Levene’s test. When an ANOVA was significant, Tukey’s honestly significant difference (HSD) test was used for *post hoc* analysis. When homogeneity of variance or normality was not achieved, means were compared using a Kruskal–Wallis non-parametric ANOVA, with Bonferroni–Dunn’s test as *post hoc* analysis. Pearson correlation coefficient and linear regression between inter-treatment and intra-population trait variability and the PPI of the different traits for each taxon were calculated to analyse the relationships between trait variability among and within taxa. Correlation (Pearson correlation coefficient) and regression (univariate linear regression) analyses were also applied to explore the association between phenotypic inheritance as dependent variable and parental trait variabilities as independent variables, calculating the relationships between the number of hybrid individuals with transgressive or parental dominated traits (dependent variable) and the variability indexes for the parental species for each trait (independent variable).

## Results

### Phenotypic inheritance

A total of 33 out of the 37 evaluated plant traits changed with salinity for at least one taxon, while 35 traits showed differences between taxa in at least one salinity treatment level **[see****Supporting Information**—**Fig. S1****]**.

The parental species showed differences in the traits measured at the different salinity treatments (ANOVA, *P* < 0.05). *Spartina densiflora* presented greater leaf size and LWC than *S. maritima* that showed higher SLA. Regarding the biochemical traits, *S. maritima* had a higher proline, C and N foliar content, and showed higher salt excretion rate than *S. densiflora* in the presence of salt (10, 20 and 40 ppt salinity). However, leaf C:N ratio of *S. densiflora* was ca. 2 times higher than that of *S. maritima.* The pigments content (chlorophylls, carotenoids and anthocyanin) of *S. maritima* exceeded that of *S. densiflora* only at hypersalinity. Differences in Chl fluorescence between both species occurred mainly at the extremes of the salinity gradient (0.5 and 40 ppt), except for the higher *F*_0_ at sunrise in all treatments and higher Φ_PSII_ at noon of *S. densiflora* at 10 ppt salinity. At hypersalinity, *S. maritima* showed higher Φ_PSII_ and *F*_v_/*F*_m_ at sunrise and *S. densiflora* greater qP at sunrise and non-photochemical quenching (NPQ) and *F*_m_ at sunrise and noon. At freshwater, *S. maritima* exhibited higher luminescence and *F*_0_ and *S. densiflora* higher *F*_m_ at noon. Regarding the gas exchange, *S. maritima* showed higher WUE at 20 ppt salinity and higher *A*_max_ at 20–40 ppt salinity than *S. densiflora*. Finally, the apical growth of *S. densiflora* was maximum at 10 ppt salinity, being always superior to the constant growth rate of *S. maritima***[see****Supporting Information—Fig. S1****]**.

**Figure 1.**
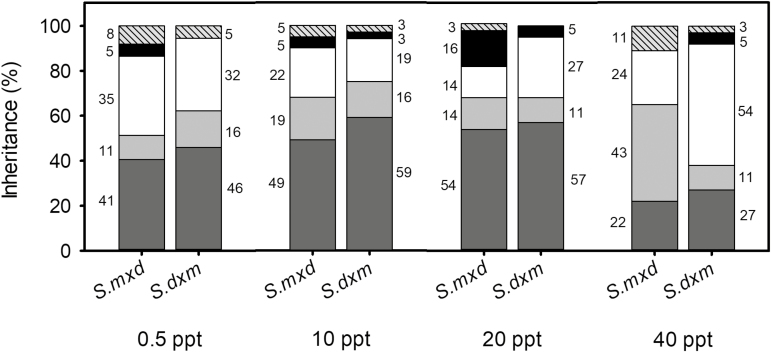
Percentage of different phenotypic inheritances for 37 foliar traits for the hybrids *Spartina maritima* × *densiflora* (*Sm*×*d*) and *S. densiflora* × *maritima* (*Sd*×*m*) at 0.5, 10, 20 and 40 ppt salinity. Inheritance types: parental codominance (dark grey); parental additivity (light grey); parental dominance of *S. densiflora* (white); parental dominance of *S. maritima* (black); transgressive (striped).

The phenotypic inheritances at the population level of both hybrids for every trait and salinity are listed **[see****Supporting Information—Table S1****]**. The traits dominated by the parental *S. densiflora* were at least 30 % greater at freshwater than at any elevated salinity concentration for *S. maritima* × *densiflora*. On the contrary, they were at least 40 % higher at hypersalinity than at the rest of the salinity levels for *S. densiflora* × *maritima*. At lower salinity concentrations (0.5 and 10 ppt), both hybrids had a similar percentage of traits dominated by *S. densiflora*, but *S. densiflora* × *maritima* exhibited a higher dominance by *S. densiflora* than *S. maritima* × *densiflora* at higher salinity ranges (20 and 40 ppt). No particular traits were dominantly characteristic of *S. maritima* for the *S. densiflora* × *maritima* hybrid at freshwater (5 % for *S. maritima* × *densiflora*) or for *S. maritima* × *densiflora* at hypersalinity (5 % for *S. densiflora* × *maritima*). The dominance of *S. maritima* trait responses was higher for *S. maritima* × *densiflora* than for *S. densiflora* × *maritima* at the intermediate treatments (5 % vs. 3 % at 10 ppt salinity; 16 % vs. 5 % at 20 ppt salinity). The traits dominated by both parents (parental codominance) were lower at the extremes of the salinity gradient than at intermediate salinities (Fig. 1).

The traits showing intermediate values ​​between both parents were ​​between 11 and 19 % for both hybrids at every salinity, except for *S. maritima* × *densiflora* at hypersalinity (43 %) (Fig. 1).

Transgressive traits were more abundant at freshwater and hypersalinity than at the intermediate salinity levels (Fig. 1). At the population level, transgressive traits for *S. maritima* × *densiflora* were leaf C:N, *A* and *A*_max_ at freshwater, NPQ (at sunrise) and maximum quantum efficiency of the photosystem II (PSII) photochemistry (*F*_v_/*F*_m_) (at noon) at 10 ppt salinity, quantum efficiency of PSII (Φ_PSII_) (at noon) at 20 ppt, and Φ_PSII_ (at noon), and *G*_s_, *C*_i_ and WUE at hypersalinity. Transgressive traits at the population level for *S. densiflora* × *maritima* were *G*_s_ and *A*_max_ at freshwater, *G*_s_ at 10 ppt and proline at hypersalinity **[see****Supporting Information—Table S2****]**. At the individual level, every hybrid showed a unique transgressive profile. Both hybrids had individuals with the same nine transgressive traits at freshwater, eight transgressive traits at 10 ppt salinity, six at 20 ppt and four at hypersalinity. Six traits (SLA, LWC and four traits related to the Chl fluorescence at sunrise) did not show any transgressive individual at any salinity. *F*_0_ and Φ_PSII_ (at noon), *A*_max_ and *G*_s_ were the traits showing more transgressive individuals (>14 individuals) **[see****Supporting Information—Fig. S2****]**.

### Trait variability

Average inter-treatment variability for every trait (*Sm*: 61 ± 4 %, *Sm*×*d*: 56 ± 5 %, *Sd*×*m*: 51 ± 4 %, *Sd*: 51 ± 4 %), average intra-population variability (*Sm*: 35 ± 4 %, *Sm*×*d*: 34 ± 3 %, *Sd*×*m*: 32 ± 3 %, *Sd*: 30 ± 3 %) and average phenotypic plasticity (*Sm*: 26 ± 2 %, *Sm*×*d*: 22 ± 2 %, *Sd*×*m*: 19 ± 2 %, *Sd*: 20 ± 2 %) were similar for all taxa (one-way ANOVA, *P* > 0.05), except for PPI being higher for *S. maritima* than *S. densiflora* × *maritima* (one-way ANOVA, *H* = 8.03, *n* = 37, *P* < 0.05). Some leaf biochemical traits (such as free proline content and salt excretion rate), some Chl fluorescence and gas exchange traits, and leaf growth were the most variable traits **[see****Supporting Information—Table S2****]**.

Inter-treatment and intra-population trait variability were positively correlated among every taxon. Additionally, PPI was also positively correlated among all taxa, except between *S. densiflora* and *S. maritima* (Table 1). Phenotypic plasticity index for initial fluorescence (*F*_0_) and *F*_v_/*F*_m_ (at noon) and *F*_0_ and maximal fluorescence (*F*_m_) (at sunrise) was higher for *S. maritima* than *S. densiflora*, and PPI for photochemical quenching (qP) (at noon) and Chl *b* content was higher for *S. densiflora* than *S. maritima***[see****Supporting Information—Table S2****]**.

**Table 1.** Pearson correlation coefficients and *P*-values for inter-treatment trait variability (Inter), intra-population trait variability (Intra) and phenotypic plasticity measured for 37 functional traits in four taxa (*Sd*×*m*, *Spartina densiflora* × *maritima*; *Sm*×*d*, *S. maritima* × *densiflora*; *Sd*, *S. densiflora*; *Sm*, *S. maritima*). Significant correlations (*P* < 0.05) are marked in bold.

	Intra (*Sd*×*m*)	Plasticity (*Sd*×*m*)	Inter (*Sm*×*d*)	Intra (*Sm*×*d*)	Plasticity (*Sm*×*d*)	Inter (*Sd*)	Intra (*Sd*)	Plasticity (*Sd*)	Inter (*Sm*)	Intra (*Sm*)	Plasticity (*Sm*)
Inter (*Sd*×*m*)	**0.913**	**0.806**	**0.85**	**0.809**	**0.544**	**0.918**	**0.903**	**0.728**	**0.699**	**0.59**	**0.418**
	**3.15E-15**	**1.79E-09**	**2.81E-11**	**1.39E-09**	**0.001**	**1.32E-15**	**2.09E-14**	**3.35E-07**	**0.000001**	**0.0001**	**0.001**
Intra (*Sd*×*m*)		**0.495**	**0.806**	**0.866**	**0.379**	**0.876**	**0.904**	**0.629**	**0.672**	**0.593**	**0.355**
		**0.002**	**1.8E-09**	**4.74E-12**	**0.021**	**1.30E-12**	**1.78E-14**	**0.00003**	**0.00001**	**0.0001**	**0.031**
Plasticity (*Sd*×*m*)			**0.642**	**0.467**	**0.611**	**0.685**	**0.613**	**0.639**	**0.515**	**0.395**	**0.377**
			**0.00002**	**0.004**	**0.0001**	**0.00001**	**0.0001**	**0.0001**	**0.001**	**0.015**	**0.022**
Inter (*Sm*×*d*)				**0.876**	**0.744**	**0.83**	**0.818**	**0.657**	**0.775**	**0.681**	**0.414**
				**1.19E-12**	**1.28E-07**	**2.13E-10**	**6.59E-10**	**0.00001**	**1.83E-08**	**0.00001**	**0.011**
Intra (*Sm*×*d*)					**0.331**	**0.85**	**0.899**	**0.575**	**0.784**	**0.75**	0.312
					**0.046**	**2.97E-11**	**3.95E-14**	**0.0002**	**9.36E-09**	**9.08E-08**	0.0603
Plasticity (*Sm*×*d*)						**0.448**	**0.355**	**0.489**	**0.431**	0.295	**0.379**
						**0.005**	**0.031**	**0.002**	**0.008**	0.0765	**0.021**
Inter (*Sd*)							**0.943**	**0.857**	**0.715**	**0.633**	**0.375**
							**2.84E-18**	**1.36E-11**	**6.66E-07**	**0.0001**	**0.022**
Intra (*Sd*)								**0.636**	**0.704**	**0.611**	**0.389**
								**0.0001**	**0.000001**	**0.0001**	**0.017**
Plasticity (*Sd*)									**0.566**	**0.519**	0.266
									**0.0003**	**0.001**	0.112
Inter (*Sm*)										**0.879**	**0.535**
										**8.03E-13**	**0.001**
Intra (*Sm*)											0.0675
											0.691

**Figure 2.**
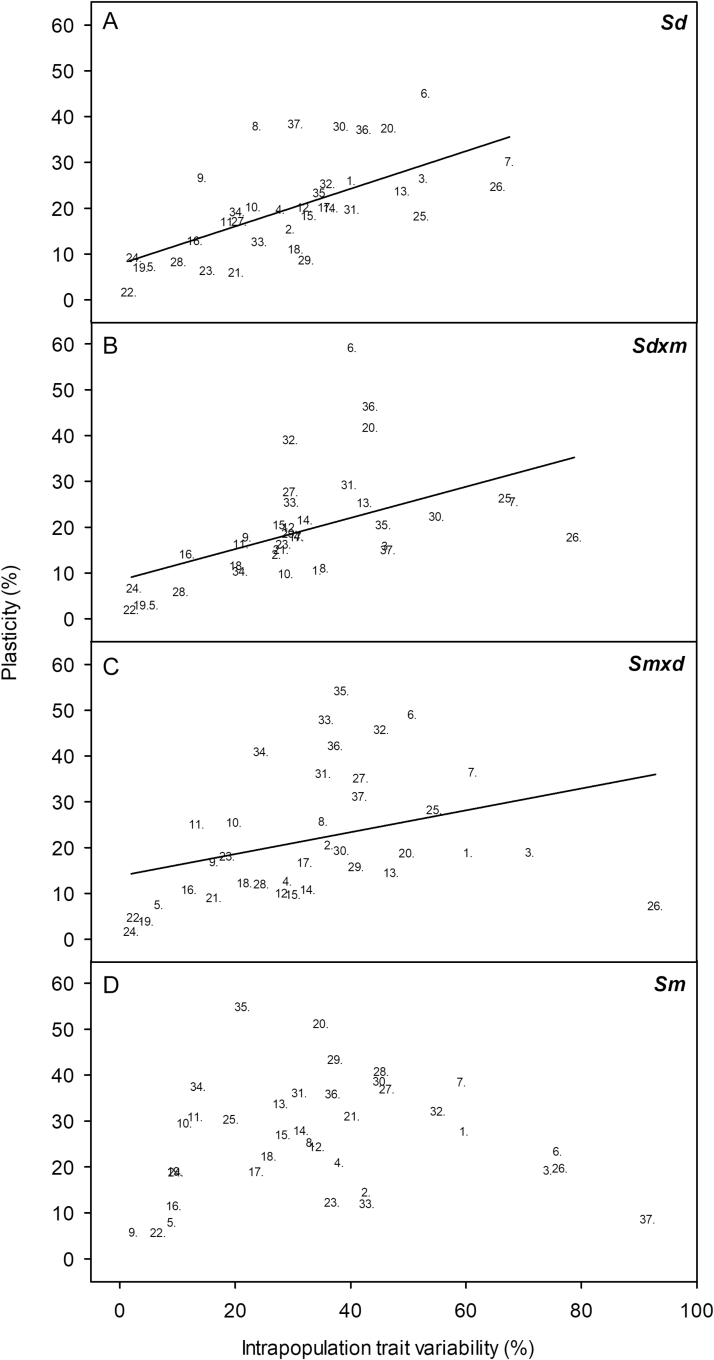
Linear regression between plasticity and intra-population trait variability for 37 foliar traits measured in *Spartina densiflora* (*Sd*; A), *S. densiflora* × *maritima* (*Sd*×*m*; B), *S. maritima* × *densiflora* (*Sm*×*d*; C) and *S. maritima* (*Sm*; D) at 0.5, 10, 20 and 40 ppt salinity. Linear regression models: *Sd*: *y* = 10.098 + 0.9964*x* (*R* = −0.64, *P* < 0.001); *Sd*×*m*: *y* = 8.574 − 1.336*x* (*R* = −0.91, *P* < 0.001); *Sm*×*d*: *y* = 13.745 + 1.242*x* (*R* = −0.87, *P* < 0.001). Traits: 1. Leaf length; 2. Leaf width; 3. Leaf area; 4. Specific leaf area; 5. Leaf water content; 6. Salt excretion rate; 7. Proline; 8. Malondialdehyde; 9. Leaf C; 10. Leaf N; 11. Leaf C:N; 12. Chl *a*; 13. Chl *b*; 14. Chl *a* + *b*; 15. Car; 16. Chl:Car; 17. Chl *a*:Chl *b*; 18. Anthocyanin; 19. qP (sunrise); 20. NPQ (sunrise); 21. *F*_0_ (sunrise); 22. *F*_v_/*F*_m_ (sunrise); 23. *F*_m_ (sunrise); 24. ΦPSII (sunrise); 25. qP (noon); 26. NPQ (noon); 27. *F*_0_ (noon); 28. *F*_v_/*F*_m_ (noon); 29. *F*_m_ (noon); 30. ΦPSII (noon); 31. Luminiscence; 32. Net photosynthesis (*A*); 33. Stomatal conductance (*G*_s_); 34. Intercellular CO_2_ concentration (*C*_i_); 35. Water use efficiency; 36. Maximum net photosynthesis (*A*_max_); 37. Leaf apical growth.

Within each taxon, inter-treatment, intra-population trait variability and PPI correlated with each other, except PPI that was independent of intra-population trait variability for *S. maritima* (Table 1); the main traits determining this lack of correlation were NPQ (at sunrise), *C*_i_ and WUE, since they showed high PPI and low intra-population variability, and leaf length, leaf area, NPQ (at noon) and leaf growth that showed low PPI and high intra-population variability (Fig. 2).

### Relationships between phenotypic inheritance and trait variability

**Figure 3.**
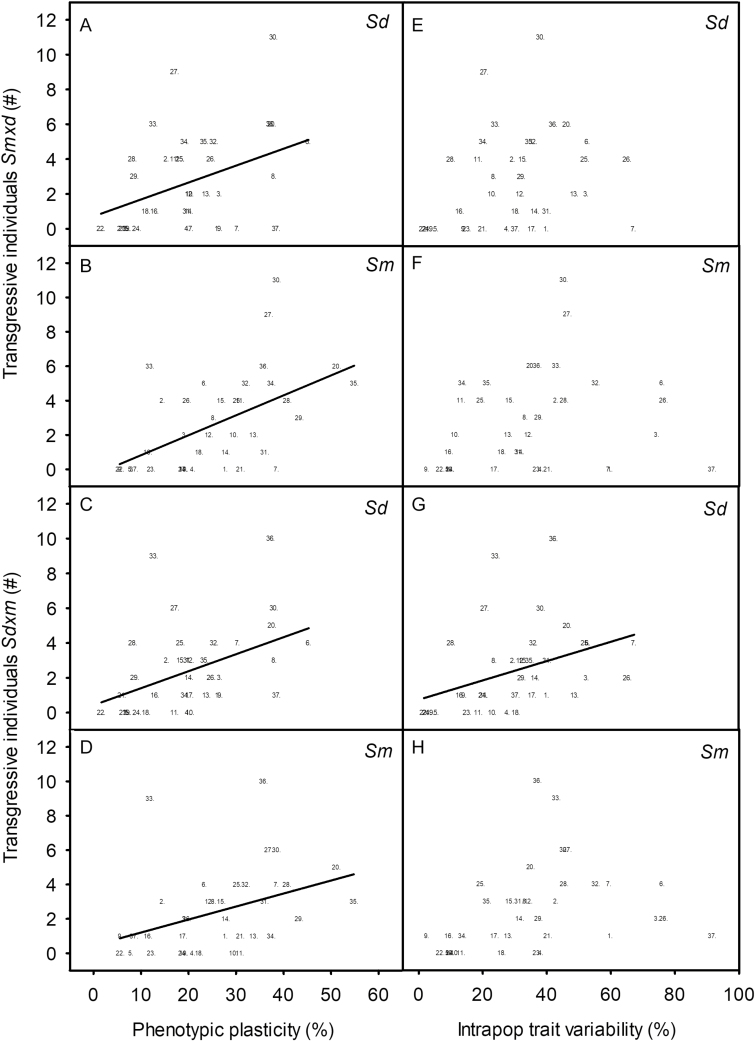
Linear regression between phenotypic plasticity and intra-population trait variability of parental species versus the number of hybrid individuals with transgressive traits for 37 foliar traits (listed in Fig. 2) measured in *Spartina maritima* (*Sm*), *S. densiflora* (*Sd*), and their hybrids *S. maritima* × *densiflora* (*Sm*×*d*) and *S. densiflora* × *maritima* (*Sd*×*m*) at 0.5, 10, 20 and 40 ppt salinity. Linear regression models: A: *y* = 0.720 + 0.0976*x* (*R* = 0.38, *P* < 0.05); B: *y* = −0.344 + 0.116*x* (*R* = −0.52, *P* < 0.001); C: *y* = 0.440 + 0.0981*x* (*R* = 0.43, *P* < 0.01); D: *y* = 0.760 + 0.0551*x* (*R* = −0.37, *P* < 0.05); G: *y* = 0.453 + 0.0756*x* (*R* = 0.38, *P* < 0.05).

The number of individuals with transgressive inheritance for a given trait for each hybrid increased together with the PPI of both parental species (Pearson correlation coefficient, *P* < 0.05, *n* = 37). On the other hand, the number of transgressive individuals for *S. densiflora* × *maritima* increased also with the intra-population trait variability of *S. densiflora*, being independent of that of *S. maritima*. The number of transgressive individuals of *S. maritima* × *densiflora* was independent of the intra-population traits variability of both parents (Table 2; Fig. 3). The lack of correlation between the number of hybrids with transgressive traits and the intra-population trait variability of their parental species were due to some highly transgressive traits with low intra-population trait variability in both parents (*F*_0_ and Φ_PSII_ (at noon), *A*_max_ and *G*_s_), and to some traits with high intra-population variability in *S. densiflora* (proline content) and *S. maritima* (leaf length, leaf area, NPQ (at noon) and leaf growth) with low number of individuals with transgressive traits for both hybrids (Fig. 3). These traits of *S. maritima* were included in those breaking the correlation between the intra-population trait variability and the PPI of *S. maritima* (Figs 2 and 3).

**Table 2.** Pearson correlation coefficients and *P*-values for intra-population trait variability (Intra) and phenotypic plasticity measured for 37 foliar traits in the parental taxa (*Sd*, *Spartina densiflora*; *Sm*, *S. maritima*) in relation with the number of hybrid (*Sd*×*m*, *S. densiflora* × *maritima*; *Sm*×*d*, *S. maritima* × *densiflora*) individuals with transgressive behaviour (T) or dominated by one of the parental species (D) for each trait. Significant correlations (*P* < 0.05) are marked in bold.

	# T (*Sd*×*m*)	# T (*Sm*×*d*)	# D-*Sd* (*Sd*×*m*)	# D-*Sd* (*Sm*×*d*)	# D-*Sm* (*Sd*×*m*)	# D-*Sm* (*Sm*×*d*)
Intra (*Sd*)	**0.373**	0.249	0.153	0.187	−0.00868	0.0117
	**0.023**	0.137	0.366	0.269	0.959	0.945
Plasticity (*Sd*)	**0.429**	**0.380**	−**0.331**	−0.231	−0.186	−0.231
	**0.008**	**0.020**	**0.046**	0.169	0.271	0.17
Intra (*Sm*)	0.321	0.197	−0.174	−0.136	0.0532	0.0735
	0.0526	0.243	0.303	0.423	0.755	0.666
Plasticity (*Sm*)	**0.377**	**0.516**	−0.04	−0.0468	−0.154	−0.169
	**0.022**	**0.001**	0.814	0.783	0.362	0.317

The number of hybrid individuals with traits dominated by any of the parents was independent of the intra-population trait variability and the PPI of each parent, except the individuals of *S. densiflora* × *maritima* with traits dominated by *S. densiflora* that decreased together with the PPI of *S. densiflora* (Table 2).

### Fitness

**Figure 4.**
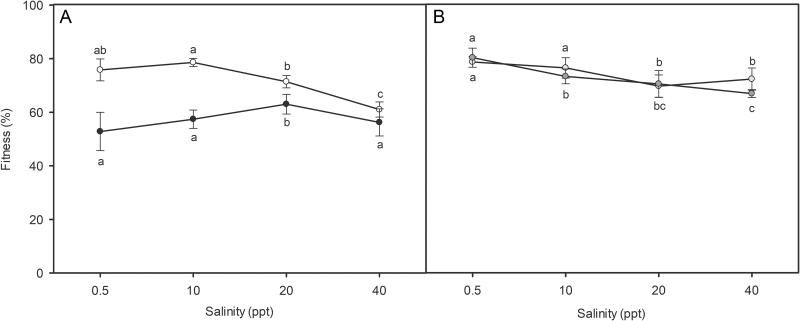
Percentage of fitness (measured as the mean of six fitness-related traits) of *Spartina maritima* ( ), *S. densiflora* ( ) (A) and their hybrids *S. maritima* × *densiflora* ( ) and *S. densiflora* × *maritima* ( ) (B) at 0.5, 10, 20 and 40 ppt salinity. Values are mean ± SD (*n* = 5). Different letters indicate significant differences between salinity treatments for the same taxon (two-way ANOVA, taxa × treatment: *F*_9, 79_ = 16.155, *P* < 0.001; Tukey’s HSD test, *P* < 0.05).

The fitness was 80 ± 1 % for *S. maritima* × *densiflora* and 76 ± 1 % for *S. densiflora* × *maritima* at freshwater (0.5 ppt salinity), being significantly higher than for the rest of the salinity treatments, except for *S. densiflora* × *maritima* at 10 ppt salinity. Fitness at 10 ppt salinity was higher than at hypersalinity (40 ppt salinity) for *S. maritima* × *densiflora* and higher than 20 ppt salinity for *S. densiflora* × *maritima*. For the parental species, the fitness of *S. densiflora* was higher at freshwater (76 ± 2 %) than at hypersalinity (61 ± 1 %) and higher at 10 ppt (79 ± 1 %) than at 20 ppt (71 ± 1 %) and 40 ppt, while *S. maritima* showed its maximum fitness at 20 ppt salinity (63 ± 2 %), being higher than at freshwater (53 ± 3 %) and hypersalinity (56 ± 2 %). *Spartina maritima* × *densiflora*, *S. densiflora* × *maritima* and *S. densiflora* showed ca. 15 % higher fitness than *S. maritima* at every treatment, except at hypersalinity. At hypersalinity, both parents showed similar fitness and lower than those of both hybrids (two-way ANOVA, taxa × salinity: *F*_9, 79_ = 8.500, *P* < 0.001; Fig. 4).

## Discussion

Our results show that the phenotypic inheritance of the studied hybrids is determined by a complex combination of different processes. Ploidy level, maternal effects and the phenotypic plasticity of the parental species are all influential processes. Also, the biochemical, physiological and morphological responses of hybrids related to their phenotypic inheritances are modulated by the abiotic environment.

### Trait variability

Inter-treatment trait variability increased together with both intra-population trait variability and phenotypic plasticity for all taxa, confirming that both are components of the former. Moreover, intra-population trait variability and phenotypic plasticity increased together for *S. densiflora* and both hybrids, so both components of trait variability seem to be regulated by the same mechanisms in these three taxa. Castillo *et al.* (2018) found the same positive relation between phenotypic plasticity and intra-population trait variability for invasive populations of *S. densiflora* in North America. Intra-population trait variability itself is the expression of the variability between the different genotypes of the population, and phenotypic plasticity may change through genetic and epigenetic mechanisms (Salmon *et al.* 2005; Crispo 2008) and therefore be inherited. In fact, both hybrids exhibited phenotypic plasticities that varied among traits like the phenotypically plasticities of both parental species, pointing to the heritability of phenotypic plasticity. On the contrary, the phenotypic plasticity of traits exhibited by the parental species did not show correlation between both taxa, reflecting independent evolution processes in response to contrasted environments since *S. maritima* was sampled from low marshes and *S. densiflora* from middle marshes. On the other hand, no relationship was found between intra-population trait variability and phenotypic plasticity for *S. maritima*, which is consistent with phenotypic plasticity being a target of natural selection that also may evolve itself with environmental changes (Pigliucci 2001). The variables that broke the correlation between intra-population variability and phenotypic plasticity for *S. maritima* were different from those breaking the correlation between the phenotypic plasticity of *S. maritima* and *S. densiflora*, showing the contrasted responses of the parental species to salinity. In this sense, it has been described that invasive taxa usually show high phenotypic plasticity that allows them to colonize highly diverse and changing environments, favouring their invasive ability (Richards *et al.* 2006; Caño *et al.* 2008; Drenovsky *et al.* 2012). Thus, high phenotypic plasticity (40 %) in response to salinity was reported for four invasive populations of *S. densiflora* from a broad latitudinal gradient along the west coast of North America (Grewell *et al.* 2016), ​similar to our inter-population trait variability (calculated the same way) for *S. densiflora* (51 %). Also, hybridization has been related to high levels of phenotypic plasticity that increases the amplitude of the niche occupied by these taxa (Ainouche and Jenczewski 2010; Te Beest *et al.* 2012; Cara *et al.* 2013). Thus, we found relatively high phenotypic plasticity for both *Spartina* hybrids and for the invasive *S. densiflora* with an ancient hybrid origin (Fortune *et al.* 2008). However, native *S. maritima* showed similar levels of phenotypic plasticity as both of its hybrids and *S. densiflora*. This may be related to the highly fluctuating salinity levels and other stress factors in the abiotic environment in their low salt marsh habitat (Contreras-Cruzado *et al.* 2017) since high environmental variability frequently leads to high adaptive phenotypic variability (Schlichting 1986; Schmid 1992; Steinger *et al.* 2003). High and heritable phenotypic plasticity was found for the stable species *Fallopia japonica* in relation to the hybrid *F.* × *bohemica*, which was attributed to epigenetic changes between clones (Parepa *et al.* 2014).

### Phenotypic inheritance

Dominant inheritance and parental additivity were recorded for both *Spartina* hybrids. *Spartina densiflora* × *maritima* showed more dominant characters from its maternal species at high salinity levels, whereas *S. maritima* × *densiflora* inherited more dominant traits from its maternal species at low salinity and also especially at 20 ppt salinity, where *S. maritima* presented its greatest fitness. Maternal effect, frequent in first-generation hybrids as those studied in this work, may affect gene expression promoting differences between hybrids that can be crucial for the divergent evolution of their tolerance to abiotic stress (Burgess and Husband 2004; Kimball *et al.* 2008; Favre and Karrenberg 2011).

Both *Spartina* hybrids showed their highest percentage of foliar transgressive traits at both extremes of the salinity gradient (freshwater and hypersalinity), whereas the trait responses resulting from the codominance of both parental species predominated at intermediate salinity levels. Transgressive traits relative to salt tolerance have also been reported for several hybrid taxa such as the hybrids between *Silene dioica* and *S. latifolia* (Favre and Karrenberg 2011) and the hybrid sunflower *Helianthus paradoxus* (Lexer *et al.* 2003).

Hybridization between native and invasive *Spartina* species has been frequent. The highly invasive and plastic allododecaploid *Spartina anglica* (2n = 12x = 120) arose after introduction of *Spartina alterniflora* (2n = 6x = 60) from the Atlantic coast of North America to European marshes. In this introduced range, it hybridized with *S. maritima* (2n = 6x = 60) and initially formed two different and independent sterile F_1_ hybrids: vigorous *Spartina* × *townsendii* (2n = 6x = 60; 2n = 9x = 90) in England (the hybrid predecessor of *S. anglica*), and *Spartina* × *neyrautii* (2n = 6x = 60) in France. Additionally, introduced *S. alterniflora* in San Francisco Bay hybridized with *Spartina foliosa* (2n = 6x = 60), native to California, forming a hybrid swarm of very plastic, invasive and fertile plants (2n = 6x = 60) (Strong and Ayres 2013). On the other hand, the ancient hybrid *S. densiflora* from South America hybridized with *S. foliosa* also in San Francisco Bay forming the reciprocal sterile hybrids *S. densiflora* × *foliosa* (2n = 6.5x = 65) and *S. alterniflora* × *foliosa* (2n = 9.5x = 95) that outperformed their parental species for different traits at extreme levels of salinity (Pakenham-Walsh *et al.* 2010). In the same way, introduced *S. densiflora* in Southwest Iberian Peninsula hybridized with *S. maritima* to form both hybrids studied in this work.

Comparing both hybrids, *S. maritima* × *densiflora* was transgressive in a greater number of foliar traits than *S. densiflora* × *maritima*, which may be related to the different ploidy level of both hybrids (*S. maritima* × *densiflora*: 2n = 95 chromosomes and *S. densiflora* × *maritima*: 2n *=* 65 chromosomes, following Castillo *et al.* 2010). A higher number of chromosomes and ploidy level usually leads to increased invasiveness through heterosis (Comai 2005; Pandit *et al.* 2014). Also, heterosis has been previously reported to be different among reciprocal hybrids with the same ploidy level, which have been related to parent-of-origin effects in *Arabidopsis* (Miller *et al.* 2012) and/or dosage effects in *Zea mays* (Yao *et al.* 2013). Transgressive traits for both *Spartina* hybrids related primarily to gas exchange and Chl fluorescence, especially the efficiencies of PSII. Ni *et al.* (2009) observed that epigenetic alterations of the circadian rhythm in allotetraploids between *Arabidopsis thaliana* and *A. arenosa* gave rise to increased photosynthetic efficiency leading to higher growth rates and heterosis.

### Parental divergence and heterosis

At the molecular level, heterosis is driven by non-additive expression of some key genes regulated genetically (dominance, overdominance and pseudo-overdominance) or epigenetically (reviewed in Chen 2013). In general, higher levels of heterosis in hybrids are found when there is a greater genetic difference between the parental taxa (East 1936; Chen 2010). For example, heterosis of *A. thaliana* crosses have been attributed to their parents showing their optimal performance at different environmental conditions that combined beneficially in the offspring (Kang 1997). *Spartina maritima* inhabits in low salt marshes, with optimum growth observed at intermediate levels of salinity (Naidoo *et al.* 2012), whereas *S. densiflora* growth is optimum at low salinity (Grewell *et al.* 2016). Hexaploid *Spartina* species (including *S. maritima*) colonize low marshes, whereas tetraploids are high marsh species (Ainouche *et al.* 2009). The heptaploid *S. densiflora* is of hybrid origin (Fortune *et al.* 2008) deriving from a hexaploid species and a (maternal) tetraploid ancestor that diverged sometimes 6–10 MYA (Rousseau-Gueutin *et al.* 2015). Additionally, the lack of correlation between the phenotypic plasticity of *S. maritima* and *S. densiflora* also suggests differentiation between the two parents, probably favouring the heterosis of their hybrids.

Results of our study goes further, demonstrating that when the parents themselves show a more plastic response for a given trait, there is a greater chance that the hybrid will develop a transgressive behaviour for this trait. This relationship seems to be mediated by epigenetic changes since both phenotypic plasticity and heterosis are regulated at an epigenetic level (Parepa *et al.* 2014) and important epigenetic changes recorded after the hybridization between *Spartina* taxa have been associated with high levels of phenotypic plasticity (Salmon *et al.* 2005; Parisod *et al.* 2009). On the other hand, the number of transgressive hybrids for a given trait in our study was independent of the intra-population trait variability of both parents, except for *S. densiflora* × *maritima* and its maternal species. Maternal effect may determine heterosis in hybrids due to maternal influence in the regulation of gene expression at transcriptional and post-transcriptional levels (Guo *et al.* 2003; Auger *et al.* 2004; Mosher *et al.* 2009). The maternal effect on transgressive traits recorded for *S. densiflora* × *maritima* was supported by a decrease in its number of traits dominated by *S. densiflora* with increasing phenotypic plasticity of *S. densiflora*.

The more frequent foliar transgressive traits of the hybrids at both extremes of the salinity gradient (freshwater and hypersalinity) coincided with their higher fitness in relation to one or both parents. Thus, both *Spartina* hybrids showed high tolerance to salt stress, presenting their maximum fitness at freshwater with a slight decrease with increasing salinity. Both hybrids exhibited higher fitness than *S. maritima* from freshwater to hypersalinity and higher than *S. densiflora* at hypersalinity. In a previous study, Naidoo *et al.* (2012) reported relationships between mechanistic physiological traits (e.g. gas exchange and Chl fluorescence) and decreases in plant fitness and overall growth of *S. maritima* in both freshwater and high salinity water. However, mechanistic foliar trait responses and overall growth of *S. densiflora* were severely limited at hypersalinity and trait responses were optimum in brackish conditions (Grewell *et al.* 2016). These studies add to the thinking that polyploidy and hybridization may lead to the expression of novel phenotypes with increased fitness (Jackson 2017).

## Conclusions

The greater tolerance of both *Spartina* hybrids to salinity than their parental species highlights the relevance of heterosis in hybridization processes. These hybrids currently maintain limited distribution in the salt marshes of the Southwest Iberian Peninsula due to their infertility (Castillo *et al.* 2010), but if a chromosomal duplication occurs in the hybrids, the new allopolyploids could become fertile (Dobzhansky 1933; Rieseberg 2001) and lead to a permanent heterosis fixation (Iehisa and Takumi 2012) increasing their capacity of invasion. Allopolyploidization has been previously documented in the genus *Spartina* for *S. anglica*, a polyploid of hybrid origin that is highly invasive (Huskins 1930; Thompson 1991; Aïnouche *et al.* 2004). Given the consequences of hybridization for increased invasiveness, the eradication of the studied *Spartina* hybrids is an urgent concern for conservation and recovery of tidal wetland plant communities.

The characteristics of the parental species that determine heterosis in their hybrids have been poorly understood. Our study reveals new data on the direct relationship between phenotypic plasticity of parents and transgressive responses of hybrids. Our results are relevant to understand the important adaptive role of interspecific hybridization in natural and potentially invasive populations. These findings in the *Poaceae* family, which include agriculturally important species such as wheat and barley, support a new focus to be applied for the artificial development of vigorous hybrid crops.

## Sources of Funding

This work was supported by a University of Sevilla research grant to B.G.T. and by the United State Department of Agriculture, Agricultural Research Service (USDAARS), Invasive Species and Pollinator Health Research Unit.

## Contributions by the Authors

The experiment was designed by B.G.T., E.F., B.J.G. and J.M.C. Greenhouse and laboratory works were performed by B.G.T., A.E.R.C., A.C. and J.M.C. All authors contributed to data analyses and writing.

## Conflict of Interest Statement

None declared.

## Supporting Information

The following additional information is available in the online version of this article—

**Methods S1.** Free proline determination.

**Methods S2.** MDA determination.

**Methods S3.** Leaf pigments determination.

**Methods S4.** Determination of chlorophyll fluorescence parameters.

**Figure S1.** Thirty-seven foliar traits for *Spartina maritima*, *S. densiflora* and their two hybrids in 0.5, 10, 20 and 40 ppt salinity. *Spartina maritima* (black); *S. maritima* × *densiflora* (dark grey); *S. densiflora* × *maritima* (light grey); *S. densiflora* (white). Values are mean ± SD (*n* = 3–5). Different letters indicate significant differences among taxa for the same salinity treatment; different numbers indicate significant differences among salinities for the same taxon (two-way analysis of variance (ANOVA), salinity × taxa, *P* < 0.05, *n* = 3–5).

**Figure S2.** Intra-population trait variability (black), phenotypic plasticity (grey) and inter-population trait variability (bar length) for 37 foliar traits measured in *Spartina maritima* (*Sm*), *S. densiflora* (*Sd*) and their hybrids *S. maritima* × *densiflora* (*Sm*×*d*) and *S. densiflora* × *maritima* (*Sd*×*m*) in 0.5, 10, 20 and 40 ppt salinity. The traits with a transgressive behaviour at the population level are marked with an asterisk.

**Table S1.** Phenotypic inheritance for the hybrids *Spartina maritima* × *densiflora* (*Sm*×*d*) and *S. densiflora* × *maritima* (*Sd*×*m*) for 37 foliar traits in six different categories: (i) leaf morphological traits, (ii) leaf biochemistry and salt excretion, (iii) pigment contents, (iv) chlorophyll fluorescence, (v) gas exchange and (vi) growth at 0.5, 10, 20 and 40 ppt salinity. Parental species: *S. maritima* (*Sm*); *S. densiflora* (*Sd*). Inheritance types: parental dominance (D); parental additivity (I); transgressive (T). The number of individuals with transgressive trait is indicated in brackets (two-way analysis of variance (ANOVA), salinity × taxa, *P* < 0.05, *n* = 3–5).

**Table S2.** Transgressive profile of *Spartina maritima* × *densiflora* (*Sm*×*d*) and *S. densiflora* × *maritima* (*Sd*×*m*) individuals (*n* = 5) at 0.5, 10, 20 and 40 ppt salinity for 37 foliar traits. Black, values over maximum values of parental species; grey, values below minimum values of parental species. The total number of transgressive individuals for a given trait and the total number of transgressive traits for a given individual are indicated.

Supporting Information
