# Supplementary material for: Phenotypic plasticity of polyploid plant species promotes transgressive behaviour in their hybrids
Source: AoB Plants. 2018 Sep 23;10(5):ply055. doi: 10.1093/aobpla/ply055 (PMC6201833; doi:10.1093/aobpla/ply055)
Supplement: Supporting Information [file ply055_suppl_supporting_information.pdf]

Phenotypic plasticity of polyploid plant species promotes transgressive behavior in their hybrids.

Authors: Blanca Gallego-Tévar, Alfredo Rubio-Casal, Alfonso de Cires, Enrique Figueroa, Brenda J. Grewell, Jesús M. Castillo

The following Supporting Information is available for this article:

Methods S1 A 0.5 g sample of fresh leaf tissue was homogenized in 10 ml of 3% sulfosalicylic acid and centrifuged at 13.000 rpm for 5 min. Two milliliters each of supernatant, acid-ninhydrin and glacial acetic acid were combined and boiled 1 h at 100 ºC in a bath. The reaction was stopped in ice and 2 ml of toluene was added to each sample. The upper toluene phase was obtained to read its absorbance at 517 nm on a spectrophotometer (Hitachi U-1900, Gemini BV, Güeldres, Netherlands) using toluene as a blank. The concentration of free proline was calculated from a standard curve of L-proline. Proportional weights and volumes were used when samples weights were lower than 0.5 g.

Methods S2 A volume of 1 ml of 0.1% trichloroacetic acid (TCA) was added to 0.2 g of ground, frozen leaf samples. The homogenate was centrifuged for 5 min at 10,000g and 4ºC. An aliquot of the supernatant (0.4-0.5 ml) was mixed with the same volume of 20% TCA, 0.5% thiobarbituric acid (TBA), 0.01% butylated hydroxytoluene. The final volume of each sample was divided into 3 sub-replicates, heated at 95ºC in a bath for 60 min, and then cooled down immediately with ice to stop the reaction. The samples were centrifuged for 5 min at 10,000 g and the absorbance of the supernatant was measured at 532 nm for determination of MDA content and the non-specific absorption at 600 nm using a spectrophotometer (GeneQuant 1300, GE Healthcare, Little Chalfont, UK). MDA concentration was calculated using its molar extinction coefficient (ε = 155 $mM^{-1}\cdot cm^{-1}$).

Methods S3 Leaf pigments were extracted using a proportion of 0.2 g of leaf FW (from one leaf per plant) and 5 ml aqueous acetone 80% and anthocyanins with a proportion of 0.2 g FW and 3 ml of 1% HCl / 80% methanol. The last extracts were incubated 24 h

at 4 ºC. All extracts were centrifuged and supernatants were used for measurement of the photosynthetic pigments chlorophyll (Chl) *a*, Chl *b* and carotenoids (Car) (mg g^{-1} FW) at 647, 664 and 470 nm in a spectrophotometer (Hitachi U-1900, Gemini BV, Güeldres, Netherlands).

Methods S4 Initial fluorescence (F_0) in the dark-adapted state was measured using a PPFD < 0.05 µmol photon m^{-2} s^{-1} for 1.8 µs, too small to induce significant physiological changes in the plant. Maximal fluorescence (F_m) was recorded after a saturating light pulse of 15.000 µmol photon m^{-2} s^{-1}. Variable fluorescence ($F_v = F_m - F_0$) and maximum quantum efficiency of the Photosystem II (PSII) photochemistry (F_v/F_m) were calculated to quantify photoinhibition. The same leaf section of each leaf was used to measure light–adapted parameters. Steady state fluorescence yield (F_s) was recorded after adapting plants to ambient light conditions (with full sunlight of 1,150 µmol photon m^{-2} s^{-1}). A saturating actinic light pulse of 15,000 µmol photon m^{-2} s^{-1} for 0.7 s was then used to produce the maximum fluorescence yield (F_m') by temporarily inhibiting PSII photochemistry. Quantum efficiency of PSII (Φ_{PSII} = (F_m' – F_s)/F_m'] was calculated. Photochemical quenching (qP) and non-photochemical quenching (NPQ) were calculated from parameters obtained in both dark and light-adapted states.

Figure S1 37 foliar traits for *Spartina maritima, S. densiflora* and their two hybrids in 0.5, 10, 20 and 40 ppt salinity. *S. maritima* (black); *S. maritima x densiflora* (dark grey); *S. densiflora x maritima* (light grey); *S. densiflora* (white). Values are mean ± SD (n = 3-5). Different letters indicate significant differences among taxa for the same salinity treatment; different numbers indicate significant differences among salinities for the same taxon (two-way ANOVA, salinity *x* taxa, $P < 0.05$, n = 3-5). Traits: **1**. Leaf length **2**. Leaf width **3**. Leaf area **4**. Specific Leaf Area (SLA) **5**. Leaf Water Content (LWC) **6**. Salt excretion rate **7**. Free proline content **8**. Malondialdehyde content **9**. Leaf Carbon **1**0. Leaf Nitrogen **11**. Leaf C : N ratio **12**. Chlorophyll (Chl) *a* content **13**. Chl *b* content **14**. Chl *a*+*b* content **15**. Carotenoids (Car) content **16**. Chl : Car ratio **17**. Chl *a* : Chl *b* ratio **18**. Anthocyanins content **19**. Photochemical quenching (qP) (sunrise) **2**0. Non-photochemical quenching (NPQ) (sunrise) **21**. Initial fluorescence (F_0) (sunrise) **22**. Maximum quantum efficiency of the Photosystem II (PSII) photochemistry (F_v/F_m) (sunrise) **23**. Maximal fluorescence (F_m) (sunrise) **24**. Quantum efficiency of PSII (ΦPSII) (sunrise) **25**. qP (noon) **26**. NPQ (noon) **27**. F_0 (noon) **28**. F_v/F_m (noon) **29**. F_m (noon) **3**0. ΦPSII (noon) **31**. Luminiscence **32**. Net photosynthesis (A) **33**. Stomatal conductance (Gs) **34**. Intercellular CO_2 concentration (Ci) **35**. Water Use Efficiency (WUE) **36**. Maximum net photosynthesis (A_{max}) **37**. Leaf apical growth.

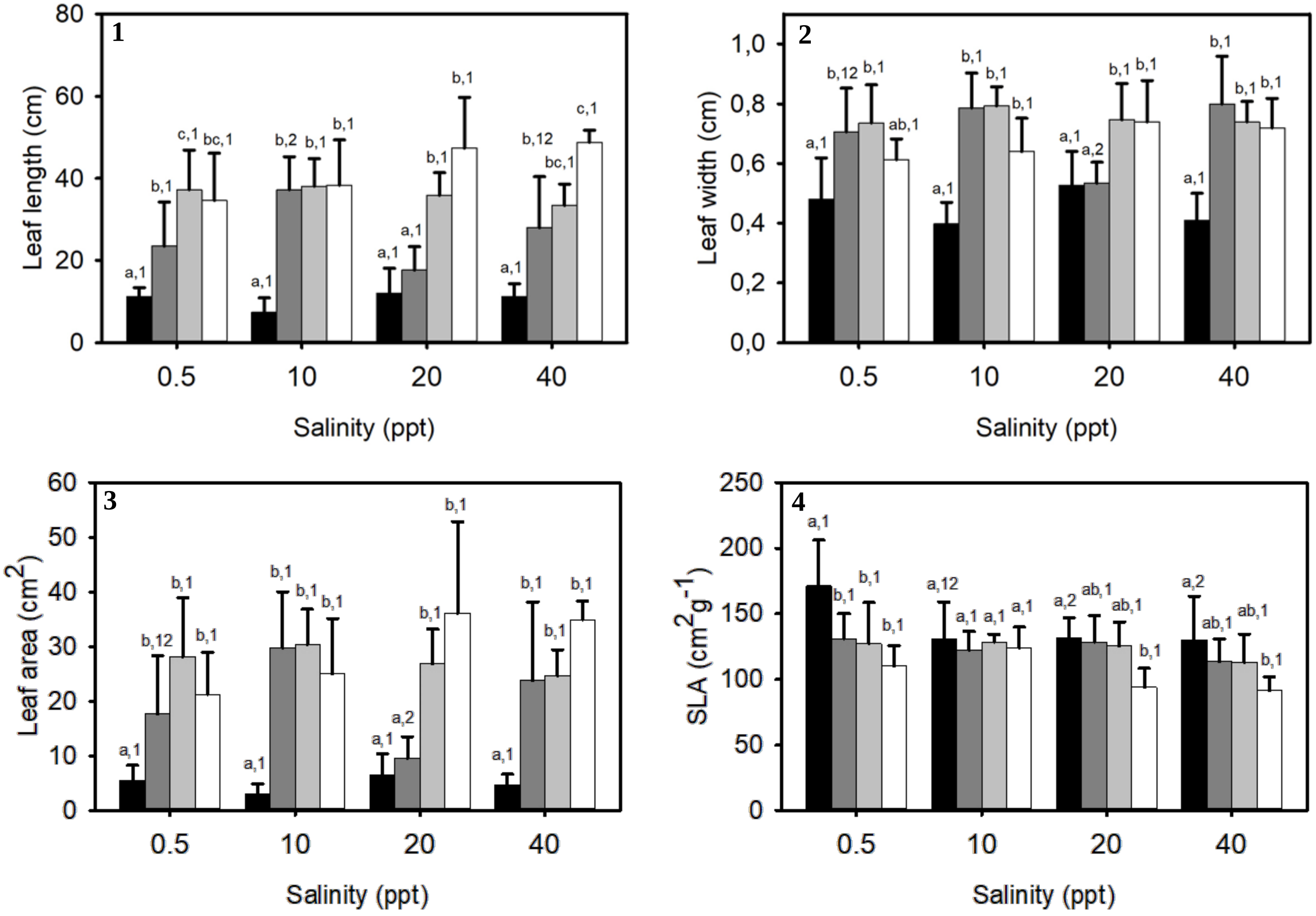
1
Leaf length (cm)
0
20
40
60
80
0.5
10
20
40
Salinity (ppt)
2
Leaf width (cm)
0,0
0,2
0,4
0,6
0,8
1,0
0.5
10
20
40
Salinity (ppt)
3
Leaf area (cm2)
0
10
20
30
40
50
60
0.5
10
20
40
Salinity (ppt)
4
SLA (cm2g-1)
0
50
100
150
200
250
0.5
10
20
40
Salinity (ppt)

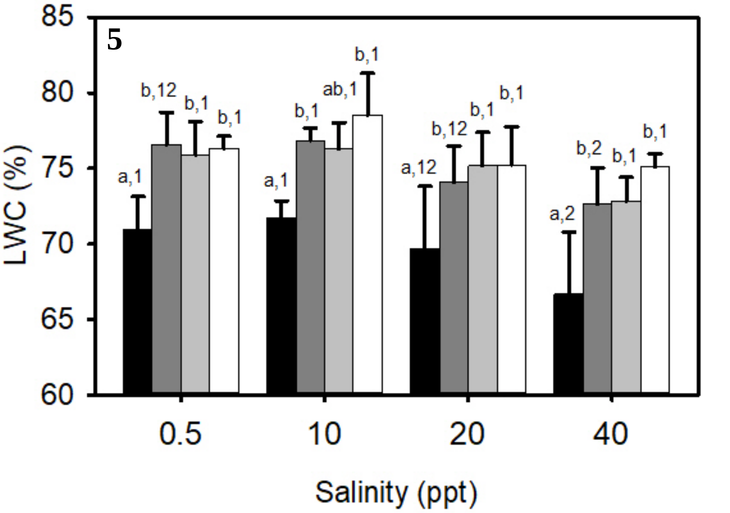
5
LWC (%)
Salinity (ppt)
0.5
10
20
40

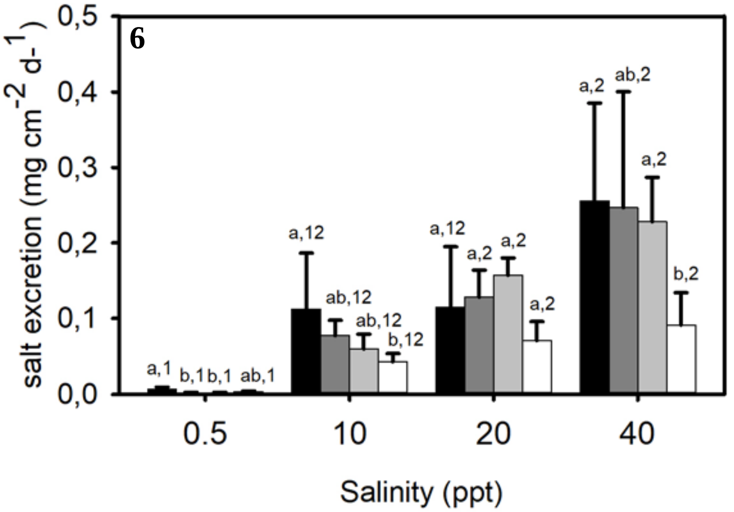
6
salt excretion (mg cm^{-2} d^{-1})
Salinity (ppt)
0.5
10
20
40

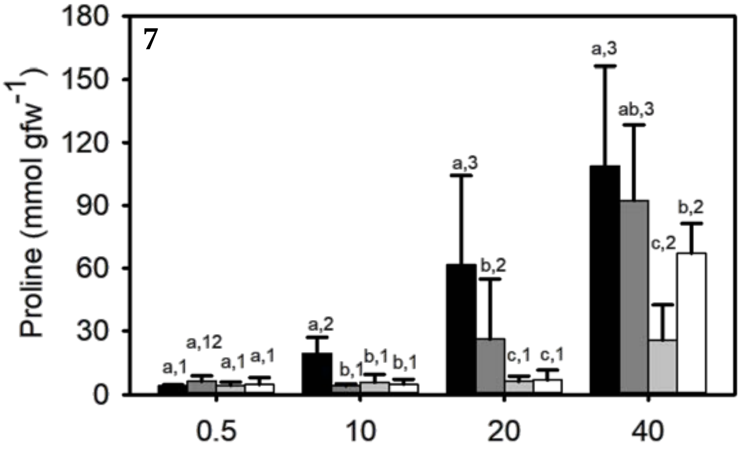
7
Proline (mmol gfw^{-1})
0.5
10
20
40

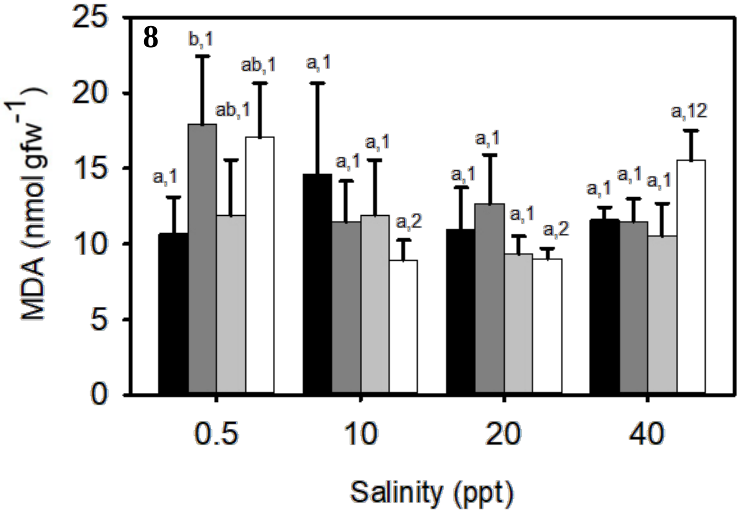
8
MDA (nmol gfw^{-1})
Salinity (ppt)
0.5
10
20
40

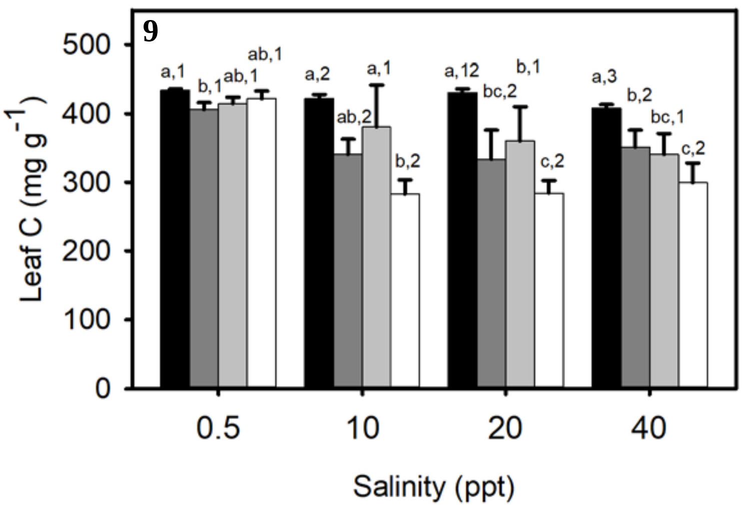
9
Leaf C (mg g^{-1})
Salinity (ppt)
0.5
10
20
40

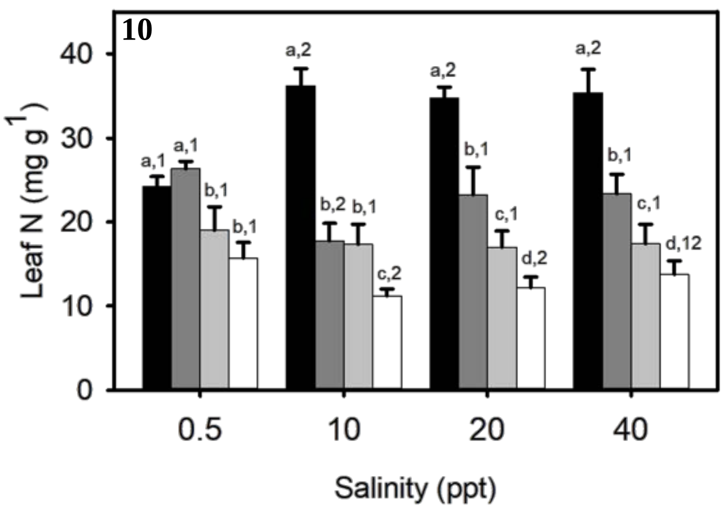
10
Leaf N (mg g^{-1})
Salinity (ppt)
0.5
10
20
40

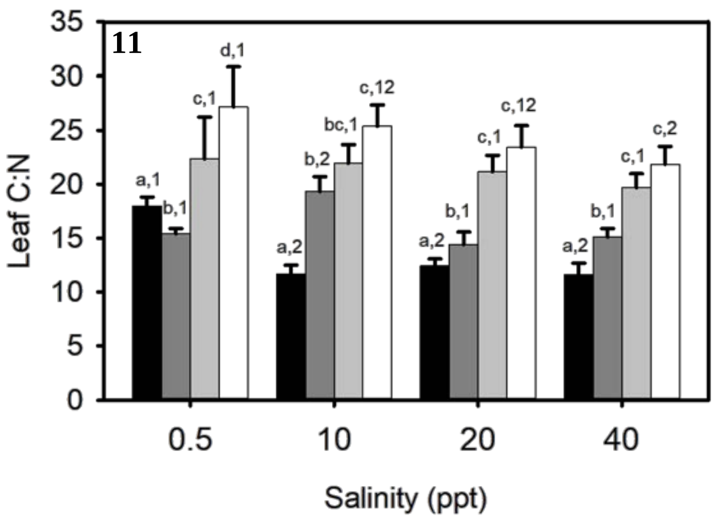
11
Leaf C:N
Salinity (ppt)
0.5
10
20
40

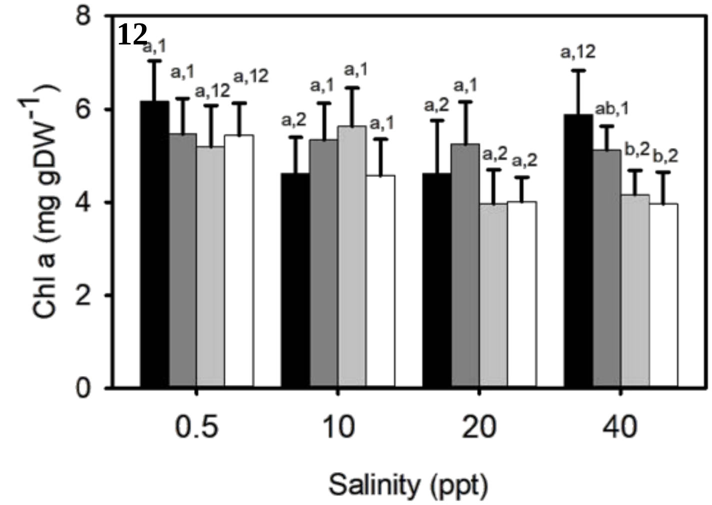
12
Chl a (mg gDW^{-1})
0.5
10
20
40
Salinity (ppt)

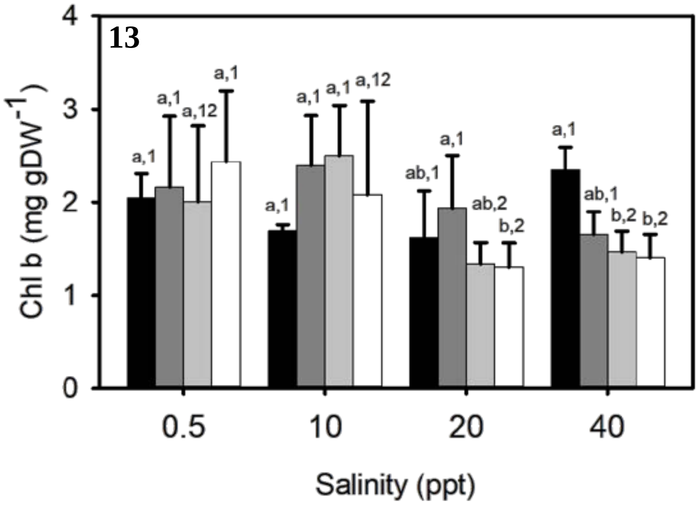
13
Chl b (mg gDW^{-1})
0.5
10
20
40
Salinity (ppt)

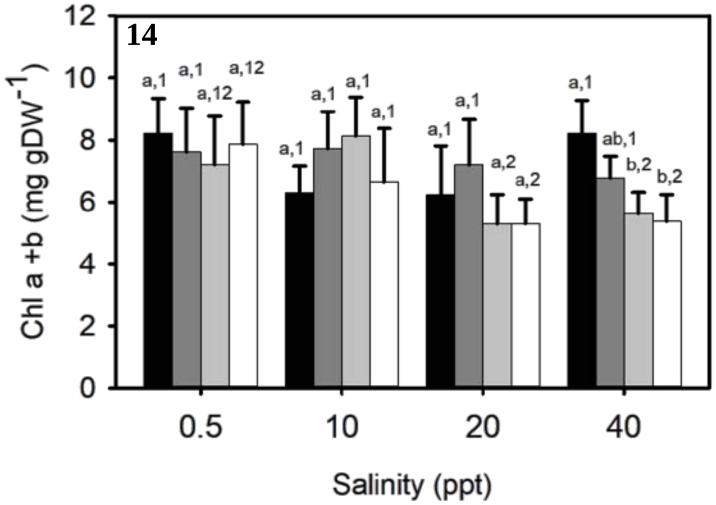
14
Chl a +b (mg gDW^{-1})
0.5
10
20
40
Salinity (ppt)

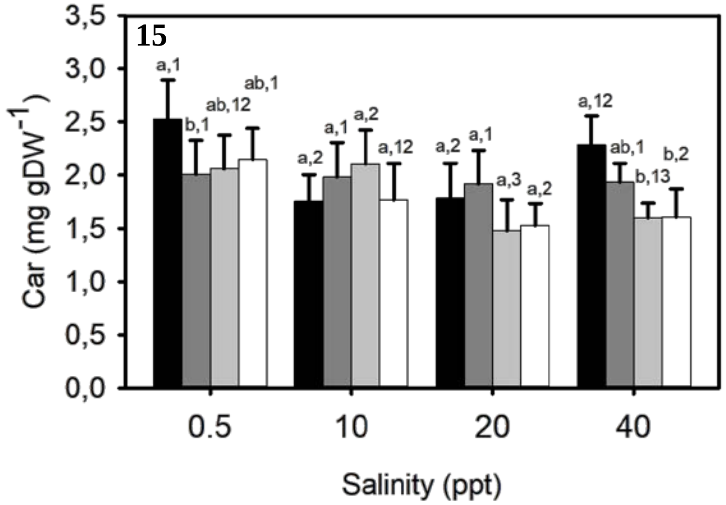
15
Car (mg gDW^{-1})
0.5
10
20
40
Salinity (ppt)

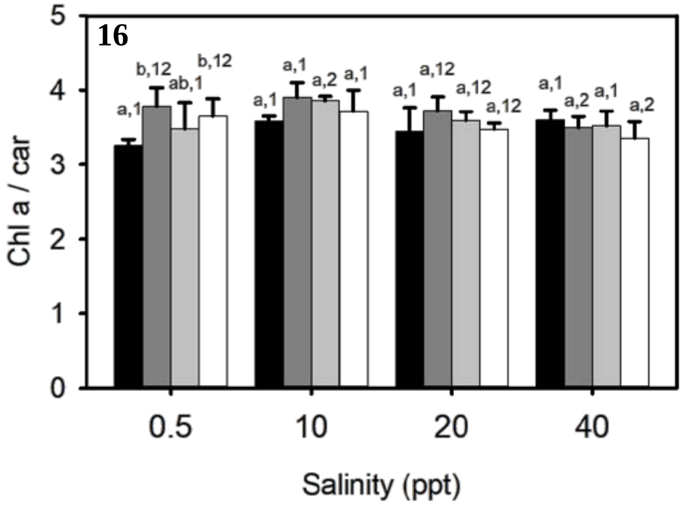
16
Chl a / car
0.5
10
20
40
Salinity (ppt)

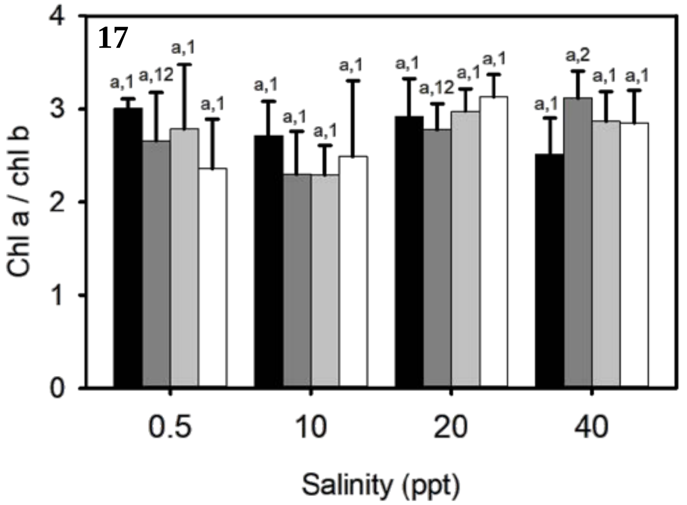
17
Chl a / chl b
0.5
10
20
40
Salinity (ppt)

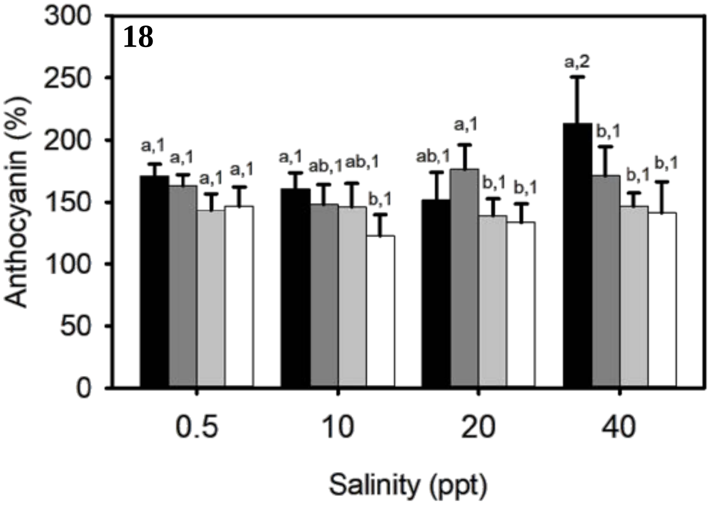
18
Anthocyanin (%)
0.5
10
20
40
Salinity (ppt)

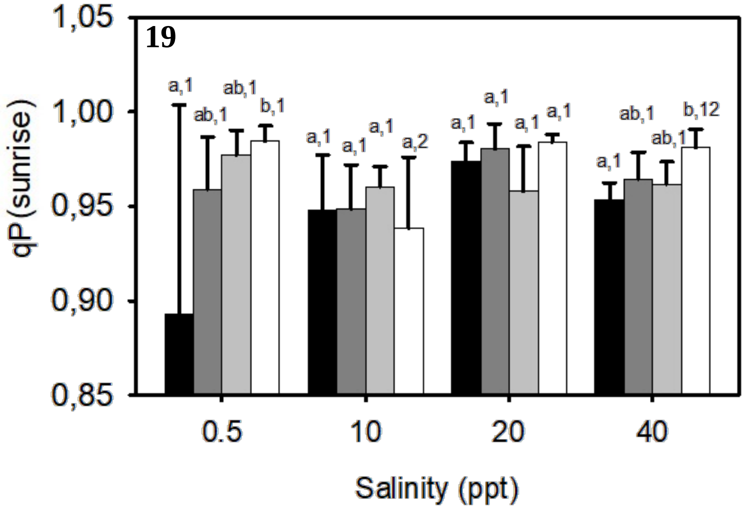
19
qP (sunrise)
1,05
1,00
0,95
0,90
0,85
a,1
ab,1
ab,1
b,1
a,1
a,1
a,1
a,2
a,1
a,1
a,1
a,1
ab,1
a,1
ab,1
b,12
0.5
10
20
40
Salinity (ppt)

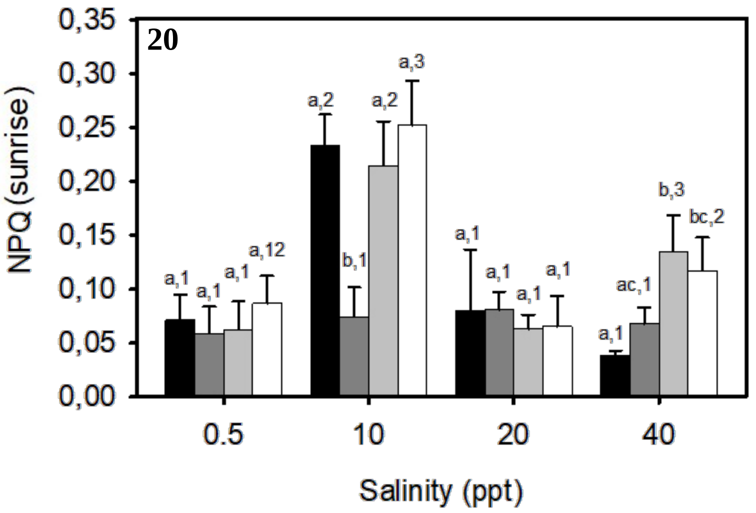
20
NPQ (sunrise)
0,35
0,30
0,25
0,20
0,15
0,10
0,05
0,00
a,1
a,1
a,1
a,12
a,2
b,1
a,2
a,3
a,1
a,1
a,1
a,1
a,1
ac,1
b,3
bc,2
0.5
10
20
40
Salinity (ppt)

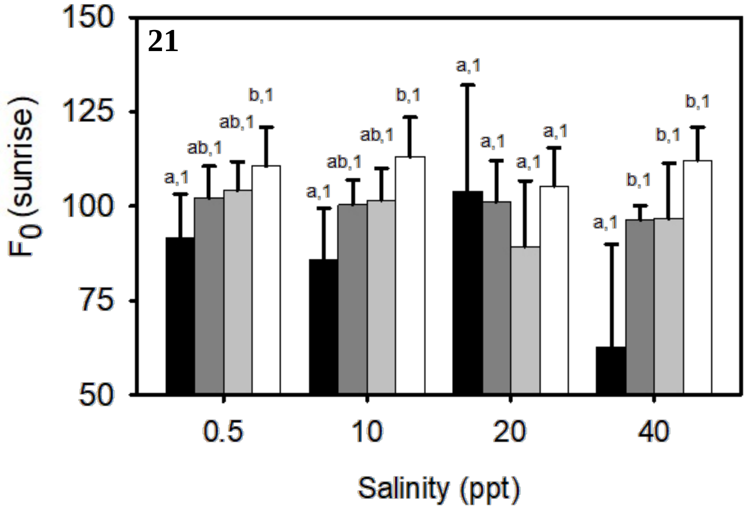
21
F0 (sunrise)
150
125
100
75
50
a,1
ab,1
ab,1
b,1
a,1
ab,1
ab,1
b,1
a,1
a,1
a,1
a,1
a,1
b,1
b,1
b,1
0.5
10
20
40
Salinity (ppt)

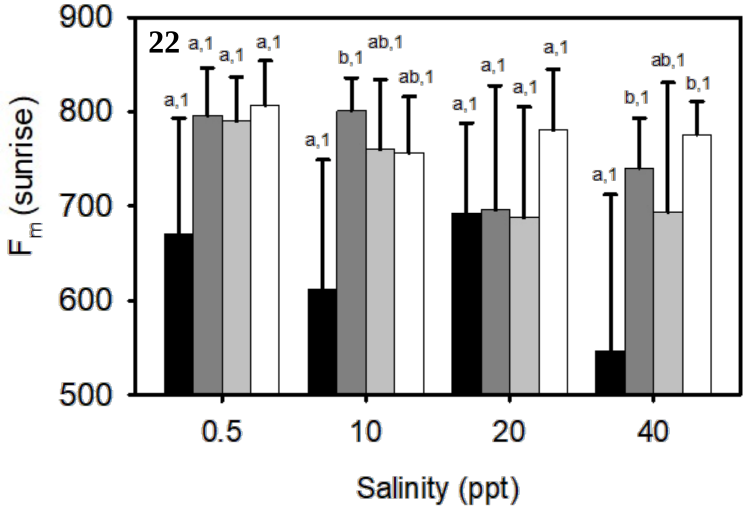
22
Fm (sunrise)
900
800
700
600
500
a,1
a,1
a,1
a,1
a,1
b,1
ab,1
ab,1
a,1
a,1
a,1
a,1
a,1
b,1
ab,1
b,1
0.5
10
20
40
Salinity (ppt)

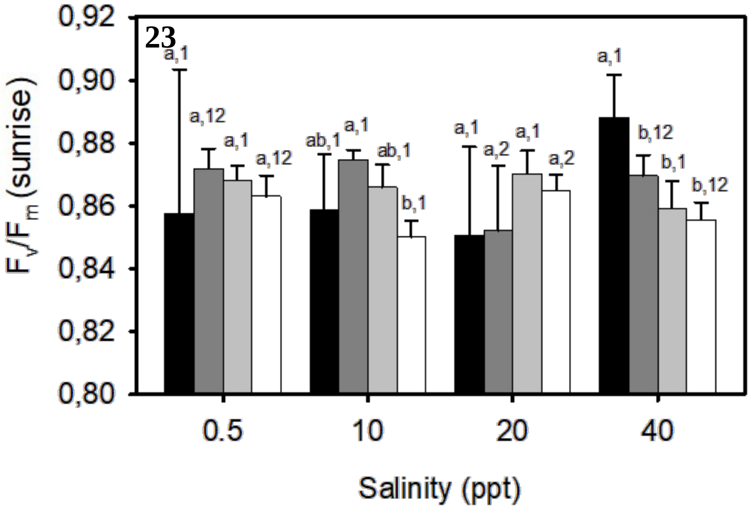
23
Fv/Fm (sunrise)
0,92
0,90
0,88
0,86
0,84
0,82
0,80
a,1
a,12
a,1
a,12
ab,1
a,1
ab,1
b,1
a,1
a,2
a,1
a,2
a,1
b,12
b,1
b,12
0.5
10
20
40
Salinity (ppt)

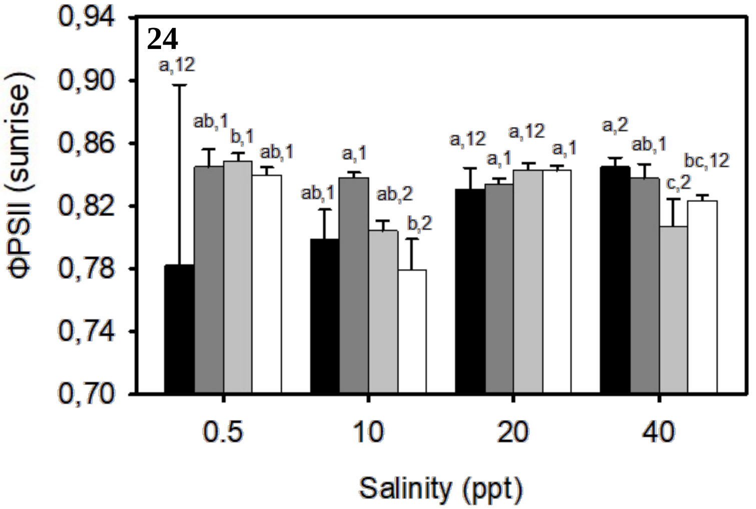
24
ΦPSII (sunrise)
0,94
0,90
0,86
0,82
0,78
0,74
0,70
a,12
ab,1
b,1
ab,1
ab,1
a,1
ab,2
b,2
a,12
a,1
a,12
a,1
a,2
ab,1
c,2
bc,12
0.5
10
20
40
Salinity (ppt)

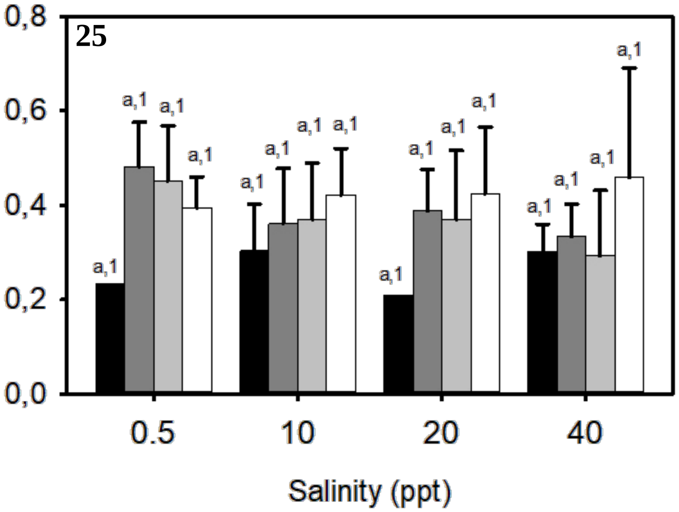
25
qP (noon)
0,0
0,2
0,4
0,6
0,8
0.5
10
20
40
Salinity (ppt)

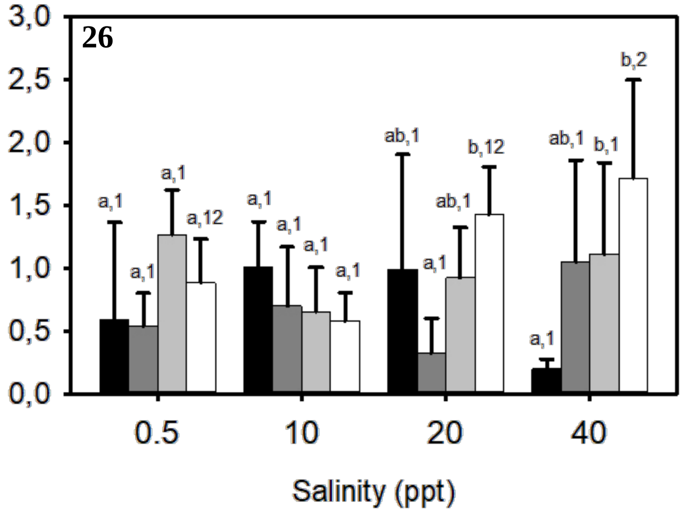
26
NPQ (noon)
0,0
0,5
1,0
1,5
2,0
2,5
3,0
0.5
10
20
40
Salinity (ppt)

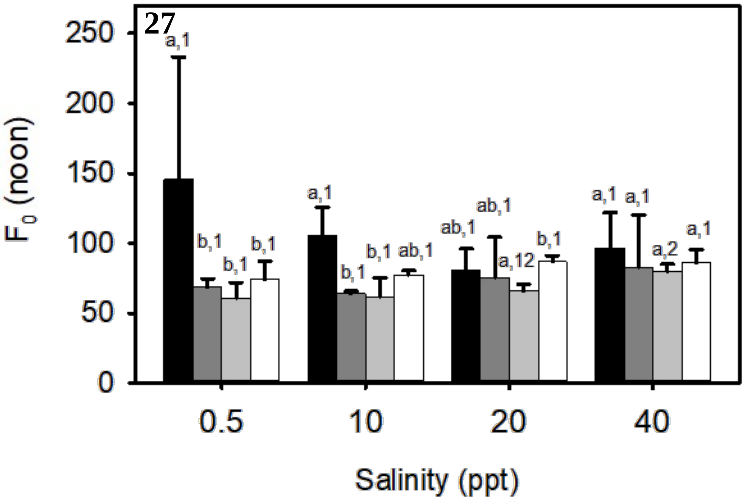
27
F_0 (noon)
0
50
100
150
200
250
0.5
10
20
40
Salinity (ppt)

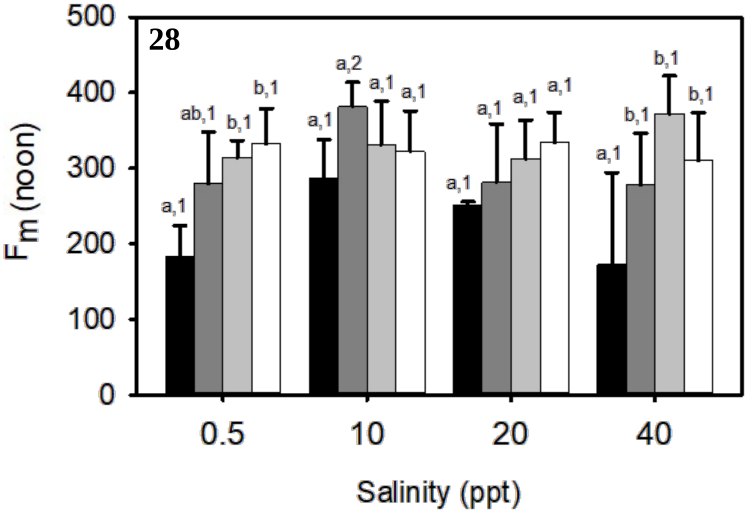
28
F_m (noon)
0
100
200
300
400
500
0.5
10
20
40
Salinity (ppt)

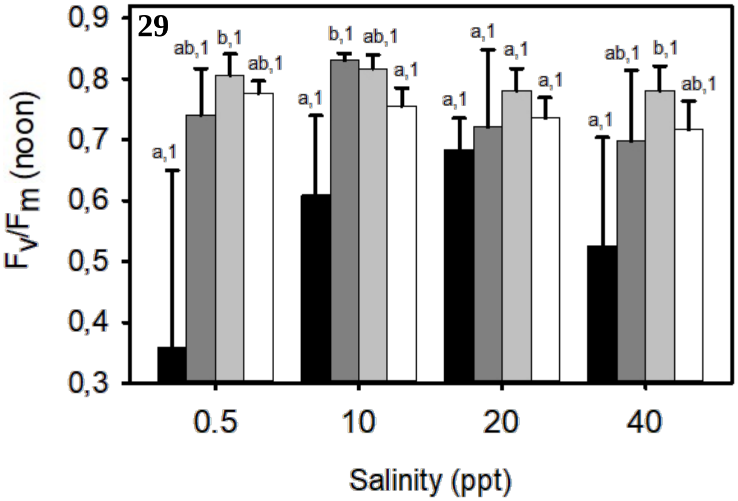
29
F_v/F_m (noon)
0,3
0,4
0,5
0,6
0,7
0,8
0,9
0.5
10
20
40
Salinity (ppt)

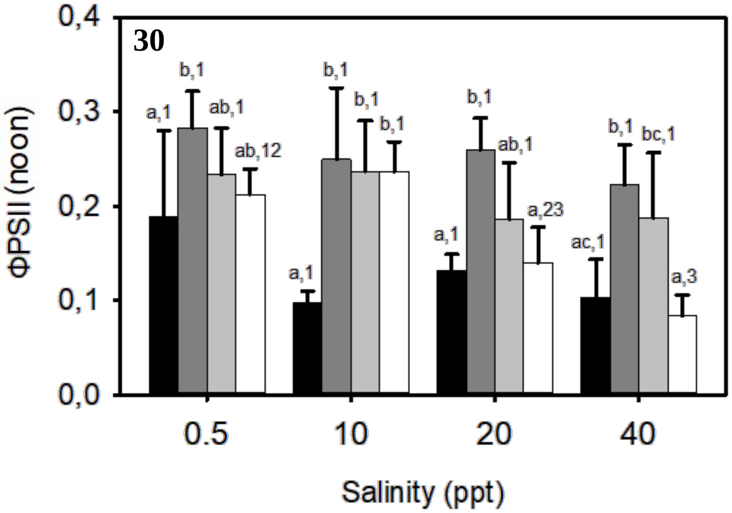
30
ΦPSII (noon)
0,0
0,1
0,2
0,3
0,4
0.5
10
20
40
Salinity (ppt)

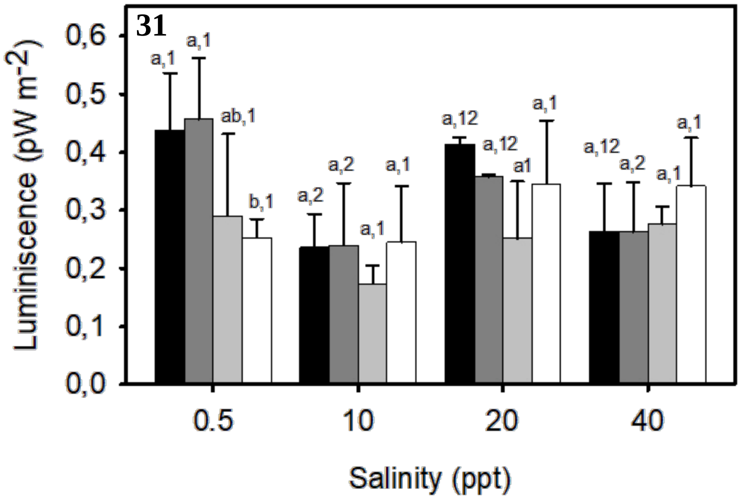
31
Luminiscence (pW m^{-2})
0,0
0,1
0,2
0,3
0,4
0,5
0,6
0.5
10
20
40
Salinity (ppt)

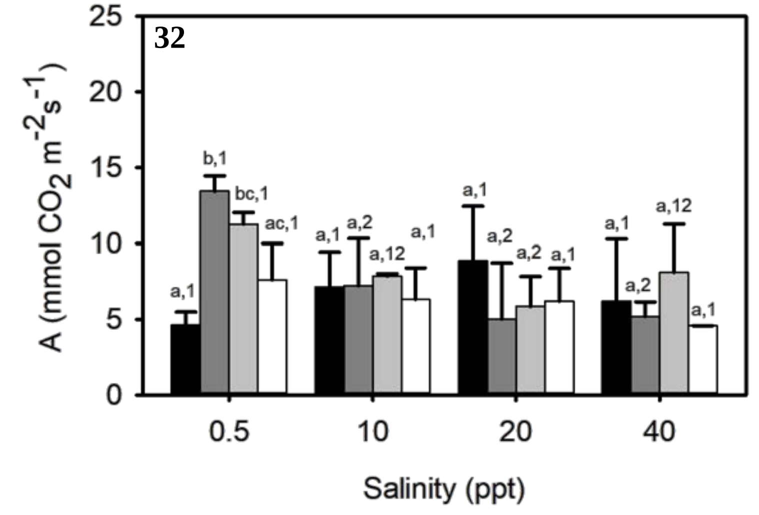
32
A (mmol CO2 m-2s-1)
25
20
15
10
5
0
0.5
10
20
40
Salinity (ppt)

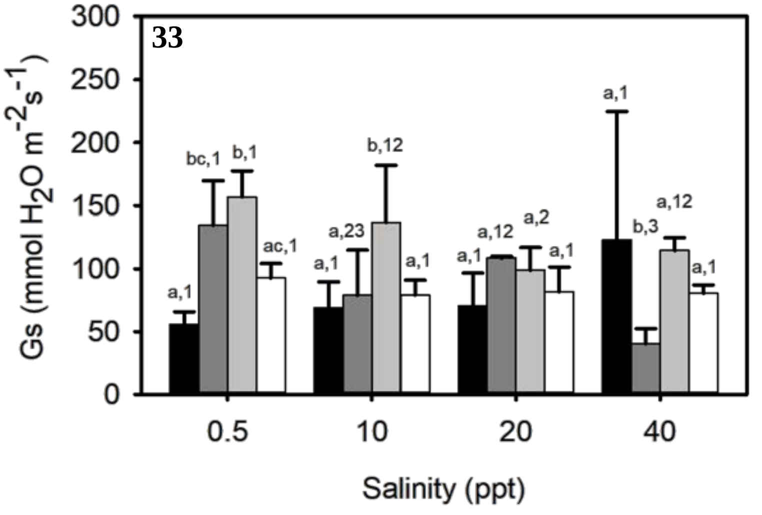
33
Gs (mmol H2O m-2s-1)
300
250
200
150
100
50
0
0.5
10
20
40
Salinity (ppt)

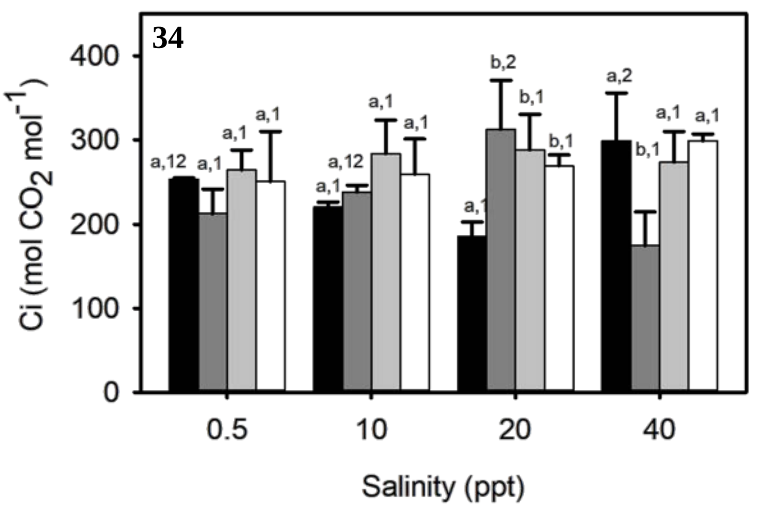
34
Ci (mol CO2 mol-1)
400
300
200
100
0
0.5
10
20
40
Salinity (ppt)

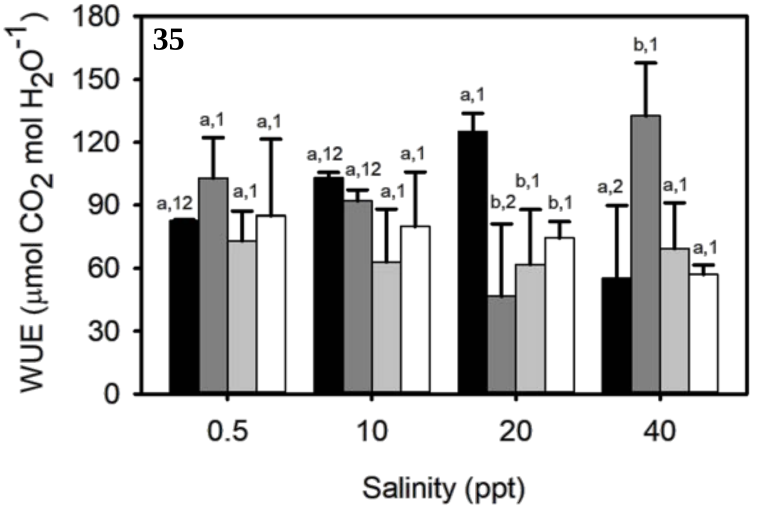
35
WUE (µmol CO2 mol H2O-1)
180
150
120
90
60
30
0
0.5
10
20
40
Salinity (ppt)

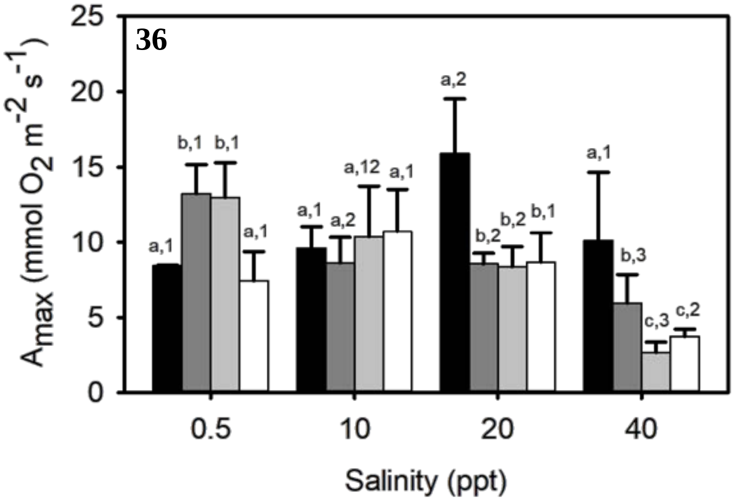
36
Amax (mmol O2 m-2 s-1)
25
20
15
10
5
0
0.5
10
20
40
Salinity (ppt)

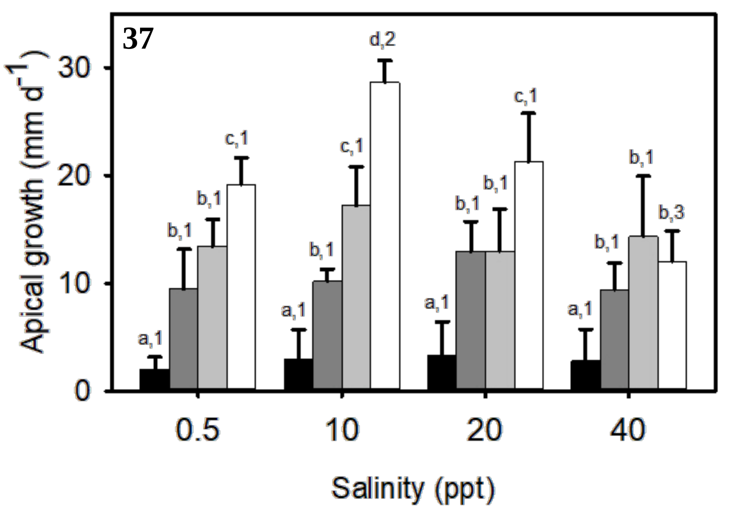
37
Apical growth (mm d-1)
30
20
10
0
0.5
10
20
40
Salinity (ppt)

Table S1 Inheritance mechanisms for the hybrids *Spartina maritima x densiflora* (*Smxd*) and *S. densiflora x maritima* (*Sdxm*) for 37 foliar traits in 6 different categories: (1) leaf morphological traits, (2) leaf biochemistry and salt excretion, (3) pigment contents, (4) chlorophyll fluorescence, (5) gas exchange, and (6) growth at 0.5, 10, 20 and 40 ppt salinity. Parental species: *S. maritima* (*Sm*); *S. densiflora* (*Sd*). Inheritance mechanisms: parental dominance (D; *Sd*, orange; *Sm*, green); parental additivity (I; white); transgressive (T; red). The number of individuals with transgressive trait is indicated in brackets (two-way ANOVA, salinity *x* taxa, *P* < 0.05, n = 3-5).

Trait / Salinity	0.5 ppt		10 ppt		20 ppt		40 ppt		ANOVA
	S.mxd	S.dxm	S.mxd	S.dxm	S.mxd	S.dxm	S.mxd	S.dxm	
**Leaf morphological traits**									
**Leaf length**	D - Sd	D - Sd (1)	D - Sd	D - Sd	D - Sm	D - Sd	I	D - Sd	Taxa, F = 74.50 P< 0.001 Treatment, n.s Interaction, F = 3.04 P< 0.001
**Leaf width**	D - Sd (1)	D - Sd (3)	D - Sd (1)	D - Sd	D - Sm	D - Sd	D - Sd (2)	D - Sd	Taxa, F = 29.07 P< 0.001 Treatment,n.s Interaction, F = 3.01 P< 0.005
**Leaf area**	D - Sd	D - Sd (2)	D - Sd (1)	D - Sd	D - Sm	D - Sd	D - Sd (1)	D - Sd	Taxa, F = 57.59 P< 0.001 Treatment, n.s Interaction, F = 3.343 P< 0.001
**SLA**	D - Sd	D - Sd	D - SdSm	D - SdSm	I	I	I	I	Taxa, F = 9.11 P< 0.001 Treatment, F = 4.18 P< 0.01 Interaction, n.s
**Some leaf contents and salt excretion**									
**LWC**	D - Sd	D - Sd	D - Sd	I	D - Sd	D - Sd	D - Sd	D - Sd	Taxa, F = 26.63 P< 0.001 Treatment, F = 10.03 P< 0.001 Interaction, n.s
**Salt excretion rate**	D - Sd (4)	D - Sd (4)	I	I	D - SdSm	D - SdSm	I (1)	D - Sm	Taxa, n.s Treatment, H = 55.88 P< 0.001
**Proline**	D - SdSm	D - SdSm	D - Sd	D - Sd	I	D - Sd	I	T (4)	Taxa, F = 21.51 P< 0.001 Treatment, F = 97.44 P< 0.001 Interaction, F = 5.45 P< 0.001
**MDA (malondialdehyde)**	D - Sd (1)	D - SdSm (1)	D - SdSm	D - SdSm	D - SdSm (1)	D - SdSm	D - SdSm (1)	D - SdSm (2)	Taxa, n.s Treatment, F = 3.55 P< 0.05 Interaction, F = 2.46 P<

									0.05
**Leaf Carbon**	D - Sd	D - SdSm	I	D - Sm (1)	D-Sd	D-Sm	I	D-Sd	Taxa, H = 29.07 P< 0.001<br>Treatment, H = 22.84 P< 0.001
**Leaf Nitrogen**	D - Sm (2)	D - Sd	I	I	I	I	I	I	Taxa, F = 264.82 P< 0.001<br>Treatment, n.s<br>Interaction, F = 15.85 P< 0.001
**Leaf C/N**	T (4)	I	I	D - Sd	I	D - Sd	I	D - Sd	Taxa, F = 226.05 P< 0.001<br>Treatment, F = 24.72 P< 0.001<br>Interaction, F = 14.26 P< 0.001
**Pigments contents**									
**Chl. a**	D - SdSm	D - SdSm (1)	D - SdSm (1)	D - SdSm (2)	D - SdSm (1)	D - SdSm	I	D - Sd	Taxa, F = 5.28 P< 0.01<br>Treatment, F = 6.99 P< 0.001<br>Interaction, F = 2.34 P< 0.05
**Chl. b**	D - SdSm (1)	D - SdSm (1)	D - SdSm	D - SdSm	D - Sm (1)	I	I	D - Sd	Taxa, n.s<br>Treatment, F = 8.56 P< 0.001<br>Interaction, F = 2.29 P< 0.05
**Chl. a + b**	D - SdSm	D - SdSm (2)	D - SdSm	D - SdSm	D - SdSm (1)	D - SdSm	I	D - Sd	Taxa, F = 3.41 P< 0.05<br>Treatment, F = 7.68 P< 0.001<br>Interaction, F = 2.49 P< 0.05
**Carotenoids**	D - Sd (2)	D - SdSm (1)	D - SdSm (1)	D - SdSm (2)	D - SdSm (1)	D - SdSm	I	D - Sd	Taxa, F = 4.85 P< 0.01<br>Treatment, F = 10.38 P< 0.001<br>Interaction, F = 2.68 P< 0.05
**Chl/Car**	D - Sd	I (1)	D - SdSm	D - SdSm	D - SdSm (1)	D - SdSm	D - SdSm	D - SdSm	Taxa, F = 5.41 P< 0.01<br>Treatment, F = 6.70 P< 0.001<br>Interaction, n.s
**Chl. a/Chl. b**	D - SdSm	D - SdSm (1)	D - SdSm	D - SdSm	D - SdSm	D - SdSm	D - SdSm	D - SdSm	Taxa, n.s<br>Treatment, H = 11.09 P< 0.01
**Anthocyanins**	D - SdSm	D - SdSm	I	I	D - Sm (1)	D - Sd	D - Sd	D - Sd	Taxa, F = 17.62 P< 0.001<br>Treatment, F = 5.74 P< 0.01<br>Interaction, F = 2.182 P< 0.01
**Chlorophyll fluorescence**									

**qP (sunrise)**	I	I	D - SdSm	D - SdSm	D - SdSm	D - SdSm	I	I	Taxa, H = 11.68 P< 0.01<br>Treatment, H = 14.50 P< 0.01
**NPQ (sunrise)**	D - SdSm (2)	D - SdSm (2)	T (4)	D - SdSm (2)	D - SdSm	D - SdSm	I	D - Sd (1)	Taxa,F = 12.94 P< 0.001<br>Treatment, F = 55.51 P< 0.001<br>Interaction, F = 8.19 P< 0.001
**Fo (sunrise)**	I	I	I	I	D - SdSm	D - SdSm (1)	D - Sd	D - Sd	Taxa, H = 24.82 P< 0.001<br>Treatment, n.s
**Fm (sunrise)**	D - SdSm	D - SdSm	D - Sd	D - SdSm	D - SdSm	D - SdSm	D - Sd	I	Taxa, F = 8.89 P< 0.001<br>Treatment, n.s<br>Interaction, n.s
**Fv/Fm (sunrise)**	D - SdSm	D - SdSm	D - Sm	D - SdSm	D - SdSm	D - SdSm	D - Sd	D - Sd	Taxa, H = 12.88 P< 0.01<br>Treatment, n.s
**PS2 (sunrise)**	D - SdSm	D - Sd	D - Sm	D - SdSm	D - SdSm	D - SdSm	I	D - Sd	Taxa, n.s<br>Treatment, F = 25.65 P< 0.001
**qP (noon)**	D - SdSm (2)	D - SdSm (1)	D - SdSm (1)	D - SdSm (1)	D - SdSm	D - SdSm	D - SdSm (1)	D - SdSm (1) (1)	Taxa, H = 9.53 P< 0.05<br>Treatment, n.s
**NPQ (noon)**	D - SdSm	D - SdSm	D - SdSm (1)	D - SdSm (1)	D - Sm (2)	D - SdSm	I (1)	D - Sd (1)	Taxa, F = 4.11 P< 0.05<br>Treatment, n.s<br>Interaction, F = 2.86 P< 0.05
**Fo (noon)**	D - Sd	D - Sd	D - Sd (5)	D - Sd (4)	D - SdSm (1) (1)	D - Sm (2)	D - SdSm (1) (1)	D - SdSm	Taxa, H = 27.23 P< 0.001<br>Treatment, n.s
**Fm (noon)**	I	D - Sd	D - SdSm (1)	D - SdSm (1)	D - SdSm (2)	D - SdSm	D - Sd	D - Sd (1)	Taxa, F = 9.83 P< 0.001<br>Treatment, F = 2.89 P< 0.05<br>Interaction, n.s
**Fv/Fm (noon)**	D - SdSm	D - Sd (1)	T (1)	D - SdSm (1)	D - SdSm (1) (1)	D - SdSm (1)	D - SdSm (1)	D - Sd (1)	Taxa, H = 35.12 P< 0.001<br>Treatment, n.s
**PS2 (noon)**	D - Sd (1)	D - SdSm	D - Sd (1)	D - Sd	T (4)	D - SdSm (2)	T (5)	D - Sm (4)	Taxa, F = 20.01 P< 0.001<br>Treatment, F = 9.07 P< 0.001<br>Interaction, F = 2.00 P= 0.055
**Luminiscence**	D - Sm	I (1)	D - SdSm (1)	D - SdSm (1)	D - SdSm	D - SdSm (1)	D - SdSm	D - SdSm	Taxa, F = 2.95 P< 0.05<br>Treatment, F = 6.11 P< 0.01<br>Interaction, n.s
**Gas exchange**									
**A**	T (3)	D - Sd (2)	D - SdSm (1)	D - SdSm	D - SdSm (1)	D - SdSm (1)	D - SdSm	D - SdSm (1)	Taxa, n.s<br>Treatment, F = 4.30 P< 0.05<br>Interaction, F = 3.04 P< 0.05

**Gs**	D - Sd (2)	T (3)	D - SdSm (1)	T (2)	D - SdSm (1)	D - SdSm (1)	T (2)	D - SdSm	Taxa, F = 6,95 P< 0.001 Treatment, n.s Interaction, F = 3.35 P< 0.01
**Ci**	D - SdSm	D - SdSm	D - SdSm	D - SdSm	D - Sd (2)	D - Sd (1)	T (3)	D - SdSm	Taxa, F = 4.31 P< 0.05 Treatment, n.s Interaction, F = 4.67 P< 0.001
**WUE**	D - SdSm	D - SdSm	D - SdSm	D - SdSm (2)	D - Sd (2)	D - Sd (1)	T (3)	D - SdSm	Taxa, F = 4.34 P< 0.05 Treatment, n.s Interaction, F = 4.78 P< 0.001
**A max**	T (5)	T (5)	D - SdSm (1)	D - SdSm (1)	D - Sd	D - Sd	I	D - Sd (4)	Taxa, F = 11.69 P< 0.001 Treatment, F = 46.63 P< 0.001 Interaction, F = 10.69 P< 0.001
**Growth**									
**Apical growth**	I	I	I	I	I	I	D - Sd	D - Sd (1)	Taxa, F = 95.69 P< 0.001 Treatment, F = 8.86 P< 0.001 Interaction, F = 4.96 P< 0.001

Figure S2 Intrapopulation trait variability (black), phenotypic plasticity (gray) and interpopulation trait variability (bar length) for 37 foliar traits measured in *Spartina maritima* (Sm)*, S. densiflora* (Sd) and their hybrids *S. maritima x densiflora* (*Smxd*) and *S. densiflora x maritima* (*Sdxm*) in 0.5, 10, 20 and 40 ppt salinity. The traits with a transgressive behavior at the population level are marked with an asterisk.

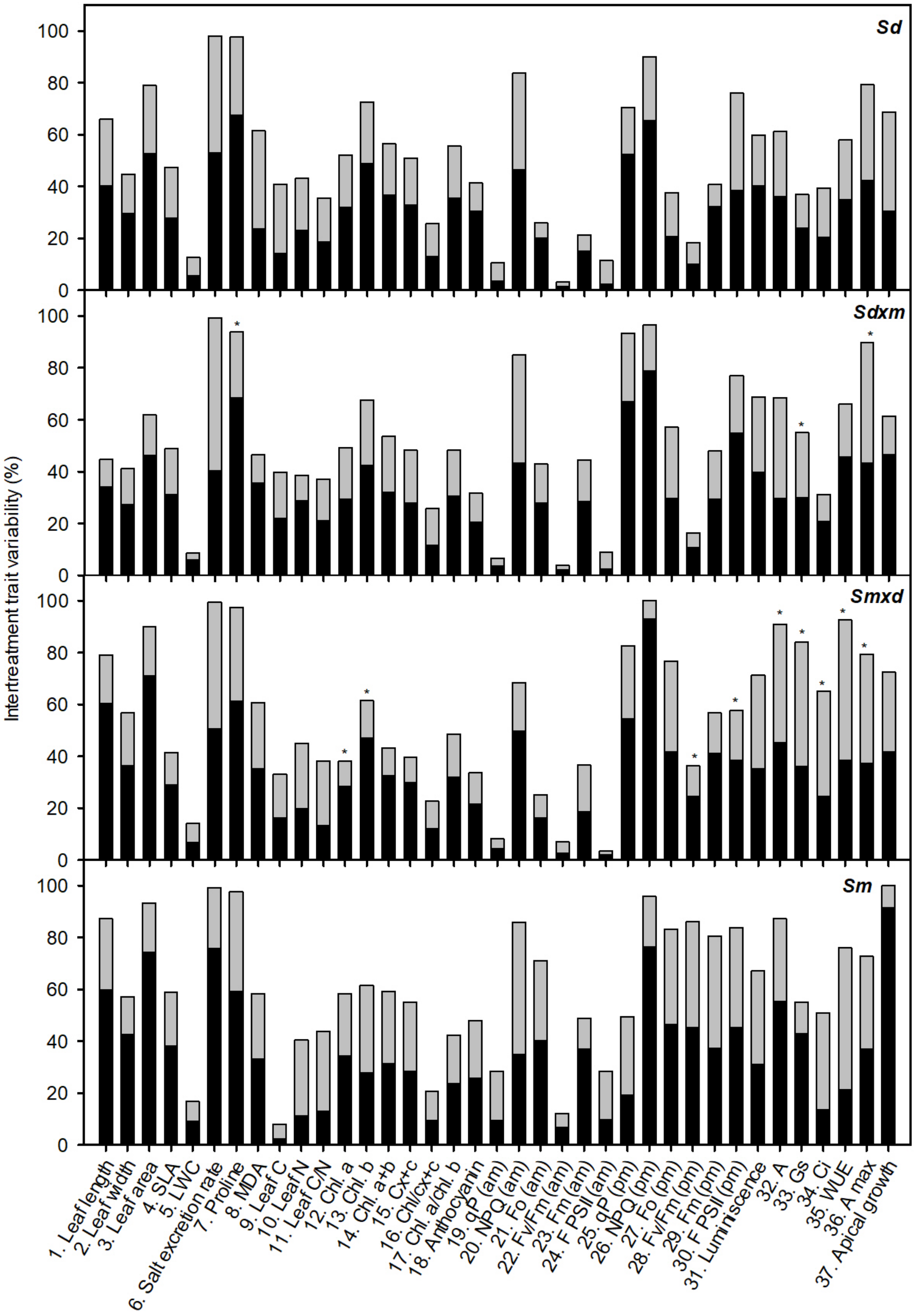

Table S2 Transgressive profile of *Spartina maritima x densiflora* (*Smxd*) and *S. densiflora x maritima* (*Sdxm*) individuals (n = 5) at 0.5, 10, 20 and 40 ppt salinity for 37 foliar traits. Black, values over maximum values of parental species; Grey, values below minimum values of parental species. The total number of transgressive individuals for a given trait and the total number of transgressive traits for a given individual are indicated.

	0.5 ppt										10 ppt										20 ppt										40 ppt										
**Trait / Individual**	*Smxd1*	*Smxd2*	*Smxd3*	*Smxd4*	*Smxd5*	*Sdxm6*	*Sdxm7*	*Sdxm8*	*Sdxm9*	*Sdxm10*	*Smxd1*	*Smxd2*	*Smxd3*	*Smxd4*	*Smxd5*	*Sdxm6*	*Sdxm7*	*Sdxm8*	*Sdxm9*	*Sdxm10*	*Smxd1*	*Smxd2*	*Smxd3*	*Smxd4*	*Smxd5*	*Sdxm6*	*Sdxm7*	*Sdxm8*	*Sdxm9*	*Sdxm10*	*Smxd1*	*Smxd2*	*Smxd3*	*Smxd4*	*Smxd5*	*Sdxm6*	*Sdxm7*	*Sdxm8*	*Sdxm9*	*Sdxm10*	Total
Leaf length									Black																																1
Leaf width					Black	Black	Black		Black			Black																				Black	Black								7
Leaf area						Black			Black			Black																					Black								4
Specific Leaf Area																																									0
Leaf Water Content																																									0
Salt excretion rate	Grey	Grey	Grey		Grey	Grey	Grey	Grey	Grey																						Black										9
Proline																																				Grey	Grey		Grey	Grey	4
Malondialdehyde				Black					Grey														Black												Grey		Grey		Grey		6
Leaf Carbon content																		Black																							1
Leaf Nitrogen content			Black	Black																																					2
Leaf C: N ratio	Grey	Grey	Grey	Grey																																					4
Chlorophyll *a* content								Grey					Black			Black		Black							Black																5
Chl *b* content			Grey				Grey																		Black																3
Chl *a*+*b*							Grey	Grey																	Black																3
Carotenoids content		Grey	Grey				Grey						Black			Black		Black							Black																7
Chl : Car ratio								Grey																	Black																2
Chl *a* : Chl *b* ratio							Black																																		1
Anthocyanin content																							Black																		1

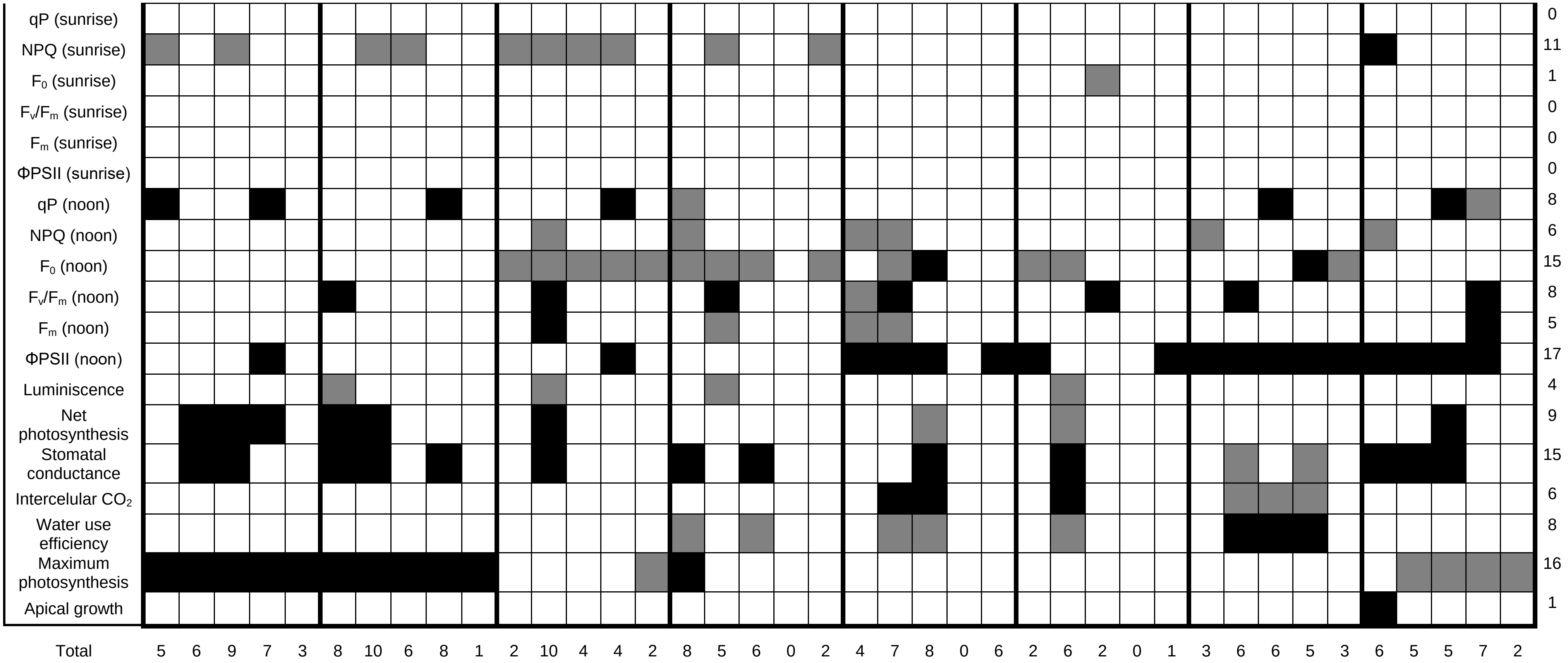
qP (sunrise)
NPQ (sunrise)
F0 (sunrise)
Fv/Fm (sunrise)
Fm (sunrise)
ΦPSII (sunrise)
qP (noon)
NPQ (noon)
F0 (noon)
Fv/Fm (noon)
Fm (noon)
ΦPSII (noon)
Luminiscence
Net photosynthesis
Stomatal conductance
Intercelular CO2
Water use efficiency
Maximum photosynthesis
Apical growth
0
11
1
0
0
0
8
6
15
8
5
17
4
9
15
6
8
16
1
Total
5 6 9 7 3 8 10 6 8 1 2 10 4 4 2 8 5 6 0 2 4 7 8 0 6 2 6 2 0 1 3 6 6 5 3 6 5 5 7 2
